# m6A RNA methylation-mediated HNF3γ reduction renders hepatocellular carcinoma dedifferentiation and sorafenib resistance

**DOI:** 10.1038/s41392-020-00299-0

**Published:** 2020-12-26

**Authors:** Tengfei Zhou, Shichao Li, Daimin Xiang, Junyu Liu, Wen Sun, Xiuliang Cui, Beifang Ning, Xiao Li, Zhuo Cheng, Weiqi Jiang, Cheng Zhang, Xijun Liang, Liang Li, Xin Cheng, Liu Hui, Hongyang Wang, Jin Ding

**Affiliations:** 1 International Cooperation Laboratory on Signal Transduction, Eastern Hepatobiliary Surgery Hospital/Institute, The Second Military Medical University, Shanghai, China; 2 Department of Gastroenterology, Changzheng Hospital, The Second Military Medical University, Shanghai, China; 3 Shanghai Institutes for Biological Sciences, Chinese Academy of Sciences, Shanghai, China; 4 The Third Department of Hepatic Surgery, Eastern Hepatobiliary Surgery Hospital, The Second Military Medical University, Shanghai, China; 5 National Center for Liver Cancer, Shanghai, China; 6 Tongji University School of Medicine, Shanghai, China

**Keywords:** Cancer, Gastrointestinal cancer

## Abstract

Hepatocyte nuclear factor 3γ (HNF3γ) is a hepatocyte nuclear factor, but its role and clinical significance in hepatocellular carcinoma (HCC) remain unclear. Herein, we report that HNF3γ expression is downregulated in patient HCC and inversely correlated with HCC malignancy and patient survival. Moreover, our data suggested that the HNF3γ reduction in HCC could be mediated by METTL14-dependent m6A methylation of HNF3γ mRNA. HNF3γ expression was increased during hepatic differentiation and decreased in dedifferentiated HCC cells. Interestingly, HNF3γ delivery promoted differentiation of not only HCC cells but also liver CSCs, which led to suppression of HCC growth. Mechanistic analysis suggested an HNF3γ-centered regulatory network that includes essential liver differentiation-associated transcription factors and functional molecules, which could synergistically facilitate HCC cell differentiation. More importantly, enforced HNF3γ expression sensitized HCC cells to sorafenib-induced growth inhibition and cell apoptosis through transactivation of OATP1B1 and OATP1B3 expression, which are major membrane transporters for sorafenib uptake. Clinical investigation showed that patient-derived HCC xenografts with high HNF3γ expression exhibited a sorafenib response and patients with high HCC HNF3γ levels benefited from sorafenib therapy. Together, these results suggest that HNF3γ plays an essential role in HCC differentiation and may serve as a therapeutic target and predictor of sorafenib benefit in patients.

## Introduction

Hepatocellular carcinoma (HCC) is one of the most prevalent malignancies in the world, and ~700,000 new cases are diagnosed annually.^1^ Surgical resection only provides an opportunity for a cure when patients are diagnosed at a very early stage. However, most patients, particular those in developing countries, are diagnosed at an advanced stage and have missed the opportunity for hepatic resection.^2^ The response of patients to conventional chemotherapy, including transcatheter arterial chemoembolization, remains disappointedly low.^3^ The complicated etiologies, including hepatitis virus infection, alcoholic liver disease, and nonalcoholic fatty liver disease, and complex pathogenesis, including chronic hepatitis, fibrosis and cirrhosis, lead to the extremely high heterogeneity of HCC, which renders it difficult to eliminate cancer cells with precision treatment or targeted drugs.^4,5^ In addition, the suppressive immune microenvironment might restrict the benefit of immune therapy in patients.^6^ Therefore, deepening the understanding of hepatocarcinogenesis and developing more effective intervention strategies for HCC is urgently needed.

Accumulating evidence has demonstrated that tumors comprise heterogeneous cell populations with different differentiation states. Tumor populations include a small portion of cancer stem cells (CSCs) and the majority of deferentially differentiated cancer cells.^7^ Compared with the more differentiated cancer cells, CSCs possess a capacity to sustain tumor growth and are more resistant to chemotherapy.^8^ Differentiation therapy aims to induce poorly differentiated cancer cells, including CSCs, into a well-differentiated benign state in which the cells largely lose their malignant phenotype.^9,10^ Differentiation therapy has been very successful in the treatment of leukemia, but its effect in solid tumors, such as HCC, is far from satisfactory.^11^

Hepatocyte nuclear factor 3 (HNF3) family, including HNF3α, HNF3β, and HNF3γ, are hepatocyte-enriched winged-helix transcription factors. HNF3α and HNF3β are the close paralogs in Foxa family members and exist widely in vertebrates. HNF3γ is only found in higher organisms, including homo sapiens.^12^ HNF3α and HNF3β have been reported to collaborate to initiate liver development while exert divergent roles in the adult liver.^13^ Okumura et al. reported that HNF3α and HNF3β but not HNF3γ dramatically repressed hepatitis B virus S protein expression in HCC cells.^14^ Kaestner et al. found that HNF3α/β protected female mice from HCC, but promoted HCC in male mice, which suggests an essential role of HNF3α and HNF3β in sexual dimorphism of liver cancer.^15^ In contrast, a recent study showed that HNF3γ was increased in lung cancer and high HNF3γ levels indicate poor patient survival.^16^ Nevertheless, the expression of HNF3γ in liver cancer and its role in HCC development remain unclear. In the present study, we clarified the expression status of HNF3γ in patient HCCs and delineated the molecular mechanism underlying its dysregulation. Moreover, the role of HNF3γ in HCC differentiation and the clinical significance of HNF3γ in sorafenib therapy were investigated.

## Results

### HNF3γ expression is downregulated in patient HCCs

**Fig. 1:**
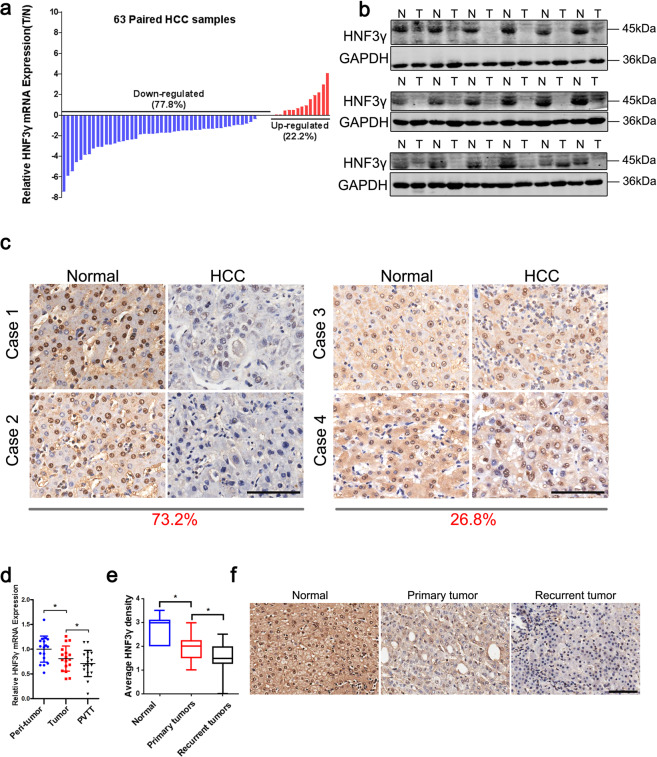
Suppressed HNF3γ expression in patient HCC tissues. **a** mRNA expression of HNF3γ in 63 pairs of HCC (T) and peri-tumoral normal (N) tissues was determined by real-time PCR. The majority of patients (77.8%) exhibited reduced HNF3γ expression in HCC tissues. **b** Western blotting assay of HNF3γ expression in 18 peri-tumoral normal (N) and paired HCC (T) tissues. **c** Representative views of HNF3γ expression in 156 paired HCC and peri-tumoral normal tissues from Cohort 1 after IHC staining. The majority of patients (73.2%) exhibited decreased HNF3γ expression in HCC tissues. Scale bar, 100 μm. **d** HNF3γ expression was compared between 17 paired normal peri-tumoral, primary HCC, and PVTT tissues by real-time PCR (*p < 0.05). **e**, **f** HNF3γ levels were compared between 21 pairs of peri-tumoral normal, primary HCC, and recurrent tumor tissues by IHC staining. Representative images are shown. Scale bar, 100 μm

Prior to investigating the role of HNF3γ in HCC, we compared the expression of HNF3γ between patient HCC and peri-tumoral normal tissues. As shown in Fig. 1a, a reduction in HNF3γ transcripts was observed in 77.8% of patient HCC samples compared with corresponding peri-tumoral normal tissues. Western blot analysis and IHC staining also revealed the decreased expression of HNF3γ in patient HCCs (Fig. 1b and Supplementary Fig. S1). Moreover, the suppressed expression of HNF3γ in HCC samples was further confirmed in a large cohort comprising 156 patients (Cohort 1) in which 73.2% of patients exhibited decreased HNF3γ expression in HCC tissues (Fig. 1c). We then examined the mRNA levels of HNF3γ in matched peri-tumoral normal tissues, primary HCCs, and portal vein thrombus tumor tissues (PVTTs) from 17 patients and found that HNF3γ expression was further reduced in metastatic HCCs (Fig. 1d). In addition, the expression of HNF3γ in recurrent HCC was much less than that in primary HCC (Fig. 1e, f). Together, these data demonstrate that HNF3γ expression is notably suppressed in HCC and might be associated with HCC progression.

### High HNF3γ levels predict a superior prognosis of HCC patients

**Fig. 2:**
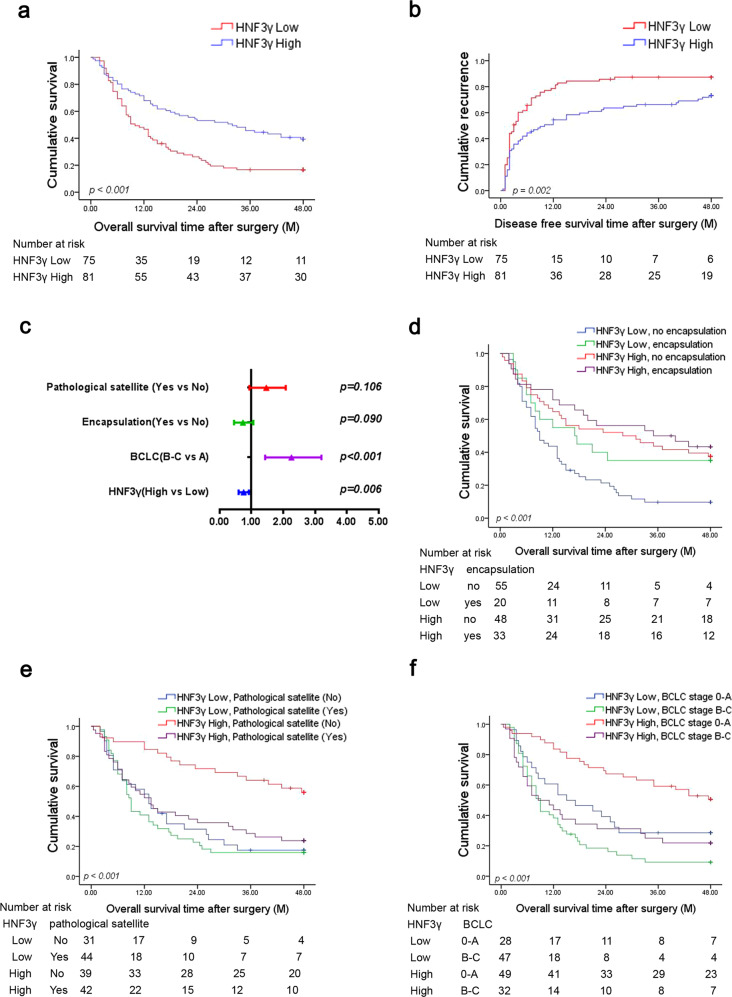
High HNF3γ levels predict a superior patient prognosis. **a**, **b** Overall survival and disease-free survival time after surgery of HCC patients were compared between the “HNF3γ low” (*n* = 75) and “HNF3γ high” (*n* = 81) groups using Kaplan–Meier analysis. **c** Multivariate analysis of overall survival of HCC patients in Cohort 1. **d**–**f** Overall survival rates of HCC patients were compared between subgroups using Kaplan–Meier analysis

Next, we analyzed the association between HNF3γ expression and patient outcome. The patients in Cohort 1 were divided into “HNF3γ Low” and “HNF3γ High” groups based on the expression levels of HNF3γ protein in their HCC samples. Pearson chi-square analysis showed that high HNF3γ levels significantly correlated with the absence of PVTT and the lower BCLC and TNM stage (Supplementary Table S1). Kaplan–Meier analysis revealed that patients in the HNF3γ High group displayed a better overall and disease-free survival than patients in the HNF3γ Low group (Fig. 2a, b). Likewise, Kaplan–Meier analysis of patient survival based on the HNF3γ mRNA levels in HCCs showed consistent results (Supplementary Fig. S2a, b). Univariate analysis showed that HNF3γ levels were correlated with patient outcome, and multivariate COX regression demonstrated that HNF3γ expression was an independent prognostic factor for patient survival (Fig. 2c and Supplementary Table S2). Furthermore, the combination of high HNF3γ levels and encapsulation, no pathological satellite, or lower BCLC stages predicted a better overall survival (OS) of patients (Fig. 2d–f). Collectively, these results indicate that HNF3γ could be a valuable predictor of patient prognosis.

### m6A RNA methylation mediates HNF3γ reduction in HCC

**Fig. 3:**
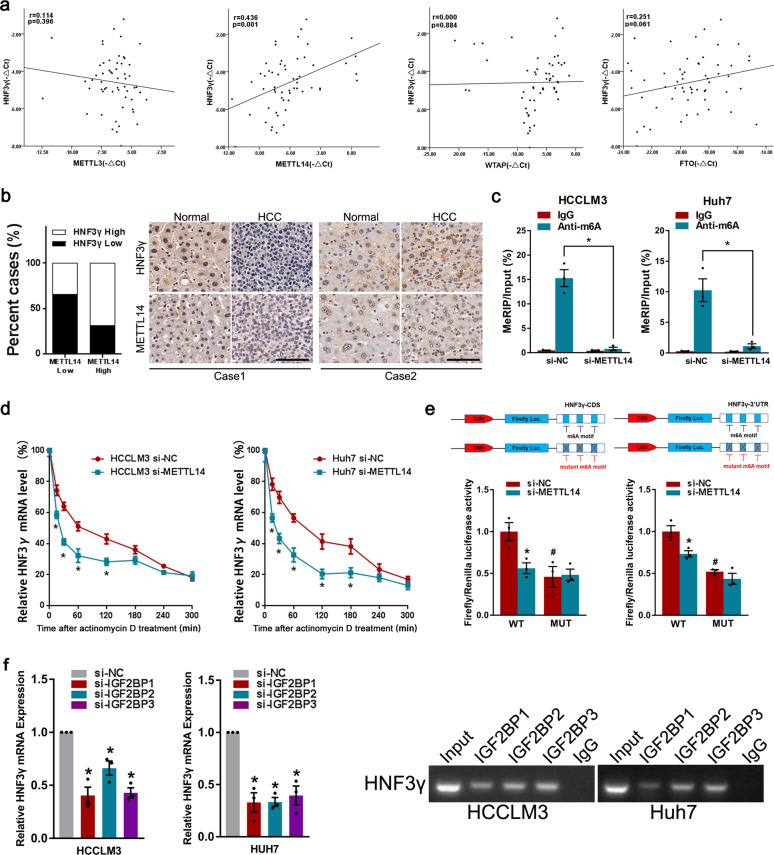
M6A analysis of HNF3γ mRNA in HCC. **a** The correlation between HNF3γ levels and METTL3, METTL14, WTAP, or FTO expression in HCC tissues was evaluated in 57 HCC specimens by Pearson’s correlation analysis. **b** The correlation between HNF3γ levels and METTL14 expression in HCC tissues from Cohort 1 was evaluated using a chi-square test. Representative IHC staining images are shown. Scale bar, 100 μm. **c** HCCLM3 and Huh7 cells were transfected with si-NC or siMETTL14 and then subjected to immunoprecipitation of m6A-modified RNA followed by real-time PCR analysis. **d** To analyze the effect of METTL14 on HNF3γ mRNA degradation, HCCLM3 or Huh7 cells transfected with si-NC or si-METTL14 were incubated with actinomycin D (10 μM) for 0, 15, 30, 60, 120, 180, 240, or 300 min. The levels of HNF3γ mRNA were determined by real-time PCR and the values were normalized to time zero. **e** Relative luciferase activity of pMIR-REPORT-HNF3γ-CDS (left panel) and pMIR-REPORT-HNF3γ-3′ UTR (right panel) with either wild-type or mutant (A-to-T mutation) m6A sites in HCCLM3 cells co-transfected with si-NC or si-METTL14, respectively. Firefly luciferase activity was measured and normalized to Renilla luciferase activity. Results were expressed as means ± SEM. * Indicates the significant difference between WT + si-NC group and WT + si-METTL14 group. # Indicates the significant difference between WT + si-NC group and MUT + si-NC group. See Supplementary materials for the details. **f** The expression of HNF3γ in HCCLM3 or Huh7 cells transfected with si-NC or si-IGF2BP1/2/3 was determined by real-time PCR. **g** RIP assay was carried out using anti-IGF2BP1/2/3 antibodies. The RNA was extracted from protein G-agarose with anti-IGF2BP1/2/3 antibody, or with control IgG. The specific anti-IGF2BP1/2/3 binding mRNA regions of HNF3γ were amplified by PCR

HNF3γ expression is suppressed in HCC and correlates with patient outcome, but the mechanism underlying the downregulation of HNF3γ remains unknown. To explore the regulatory mechanism underlying HNF3γ reduction, we treated HCC cells with a DNA methylation inhibitor or HDAC inhibitor. As shown in Supplementary Fig. S3a, b, our data suggested that HNF3γ expression was not influenced by DNA methylation or histone acetylation. The interference of Dicer, an enzyme controlling the microRNA processing, also failed to alter the expression of HNF3γ in HCC cells (Supplementary Fig. S3c). Interestingly, we found that inhibition of m6A methyltransferase such as METTL3, METTL14, or WTAP remarkably decreased the expression of HNF3γ, and knockdown of the m6A demethylase FTO but not ALKBH5 significantly increased the expression of HNF3γ (Supplementary Fig. S3d, e), suggesting that m6A modification could be involved in the HNF3γ reduction in HCC cells. As shown in Supplementary Fig. S3f, MeRIP-qPCR data suggested that m6A level of HNF3γ was lower in patient HCCs than that in paracancerous tissues. Notably, a correlation between HNF3γ expression and the levels of METTL14 but not METTL3, WTAP, or FTO was observed via real-time PCR in a group of 57 patient HCCs (Fig. 3a). Moreover, the expression of METTL14 was examined in HCC tissues of patients from Cohort 1, and a positive correlation (chi-square test, *p* < 0.05) between HNF3γ expression and METTL14 levels was observed (Fig. 3b). Since METTL14 has been reported to be reduced in patient HCCs, we speculate that METTL14 reduction could be involved in the downregulation of HNF3γ in HCCs.^17^ In further studies, a RIP assay validated the m6A modification of HNF3γ mRNA in HCC cells and confirmed the role of METTL14 in m6A modification of HNF3γ mRNA (Fig. 3c). The mRNA degradation rate of HNF3γ was then monitored at multiple time points after actinomycin D treatment and the results suggested that METTL14 could influence the stability of HNF3γ mRNA (Fig. 3d). To further elucidate the effect of m6A modification on HNF3γ mRNA stability, we performed luciferase reporter and mutagenesis assays. Our data suggested that mutation of the m6A motifs led to the decrease of HNF3γ stability (Fig. 3e). As expected, METTL14 knockdown decreased the luciferase activity in WT group but not in MUT group, further supporting the importance of m6A modification in HNF3γ mRNA stability (Fig. 3e). To identify the m6A reader required in m6A modification-regulated HNF3γ mRNA stability, we knocked down the m6A readers involved in RNA stability in HCC cells respectively.^18^ Our data showed that the interference of IGF2BP1, IGF2BP2, or IGF2BP3 (Fig. 3f and Supplementary Fig. S3g) but not YTHDF2, YTHDF3, or YTHDC2 (data not shown) resulted in the significant decrease of HNF3γ mRNA in HCC cells. Furthermore, RNA immunoprecipitation assay suggested the recognition and binding of IGF2BPs with HNF3γ mRNA in HCC cells (Fig. 3g), supporting that IGF2BPs could be involved in the stabilization of m6A-modified HNF3γ mRNA.

### HNF3γ delivery promotes the differentiation of HCC cells

**Fig. 4:**
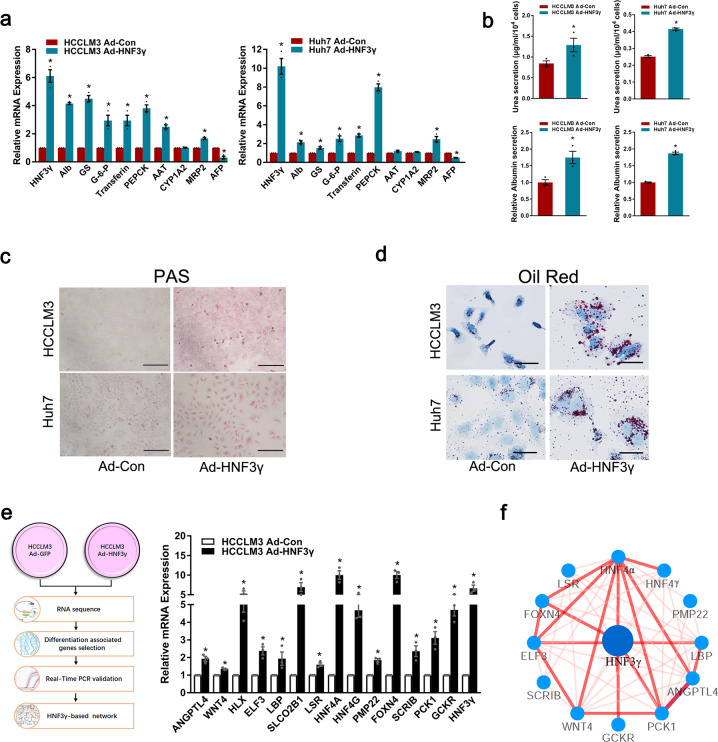
HNF3γ promotes the differentiation of HCC cells. **a** The expression of hepatocyte-specific genes and AFP in HCCLM3 or Huh7 cells infected with Ad-HNF3γ or Ad-Con was determined by real-time PCR. **b** Urea concentration in the supernatant of HCCLM3 or Huh7 cells was measured using a QuantiChrom^TM^ Urea Assay Kit (upper). The ALB secretion ability of HCC cells was measured as indicated using a Human Albumin ELISA Quantitation kit (lower). Results were expressed as means ± SEM. **c** The glycogen storage ability of HCCLM3 or Huh7 cells was evaluated by Periodic-Acid Schiff (PAS) staining. Scale bars, 100 mm. **d** The lipid accumulation ability of HCCLM3 or Huh7 cells was examined by oil red O staining (Sigma-Aldrich). Scale bars, 400 mm. **e** Hepatocyte differentiation-associated genes differentially expressed in HNF3γ-overexpressing HCCLM3 cells were obtained by RNA-seq and validated by real-time PCR. **f** HNF3γ-centered regulatory network that includes 12 liver differentiation or metabolism-associated transcription factors and functional molecules. The thickness and brightness of lines indicate the correlation degree between distinct molecules

HNF3γ has been reported to play an important role in the metabolism of mature hepatocytes, but little is known about its function in liver development and HCC differentiation. Using our in vitro hepatic differentiation system,^19,20^ we found that HNF3γ expression was notably increased during hepatic differentiation of embryonic stem cells toward hepatoblasts (Supplementary Fig. S4a), suggesting the correlation of HNF3γ and hepatic differentiation. We also examined the expression of HNF3γ in fetal liver, adult liver, and HCC tissues and found that HNF3γ expression was significantly activated in mature normal liver tissues (Supplementary Fig. S4b, c), suggesting that HNF3γ expression might be associated with the differentiation status of hepatocytes. We then investigated whether HNF3γ could facilitate the differentiation of HCC cells. As shown in Fig. 4a and Supplementary Fig. S5a, b, adenovirus-mediated HNF3γ delivery in HCC cells significantly increased the expression of hepatic function-related genes and decreased the expression of AFP. As expected, interference of METTL14 in HCC cells decreased the expression of hepatic function-related genes and increased AFP expression (Supplementary Fig. S5c). Morphological change could be observed in some HCC cells with HNF3γ overexpression (Supplementary Fig. S5d). Moreover, Ad-HNF3γ-infected HCC cells exhibited enhanced secretion of urea and albumin compared with control cells (Fig. 4b). Consistently, HCC cells infected with Ad-HNF3γ exhibited improved glycogen storage and some of which displayed enhanced lipid accumulation (Fig. 4c, d). To identify HNF3γ-regulated genes in HCC cells, RNA-seq analysis of HCC cells infected with Ad-HNF3γ or Ad-Con was conducted (Fig. 4e and Supplementary Fig. S5e). Our data revealed 30 potentially upregulated hepatic differentiation-associated genes by HNF3γ, which include liver development-related genes, cell differentiation-related genes, and hepatic metabolism-related genes (Supplementary Table S3). Fourteen of these genes, directly or indirectly upregulated by HNF3γ, were validated by real-time PCR assay (Fig. 4e and Supplementary Fig. S5f). Through bioinformatics analysis, an HNF3γ-centered regulatory network consisting of 12 of these genes that could synergistically contribute to HNF3γ-mediated HCC differentiation was constructed (Fig. 4f). ChIP assay revealed that HNF3γ could directly upregulate some of these genes with putative HNF3γ binding site in their promoter regions (Supplementary Fig. 5g). In addition, expression of HNF4α, a major downstream gene of HNF3γ in HNF3γ-centered regulatory network, was investigated and the results confirmed that HNF4α should be a downstream gene of HNF3γ in HCC cells (Supplementary Fig. S5h). Moreover, our results showed that interference of HNF4α significantly impaired HNF3γ’s tumor suppressor function in HCC cells (Supplementary Fig. S5i–k).

### HNF3γ promotes the differentiation of liver CSCs

**Fig. 5:**
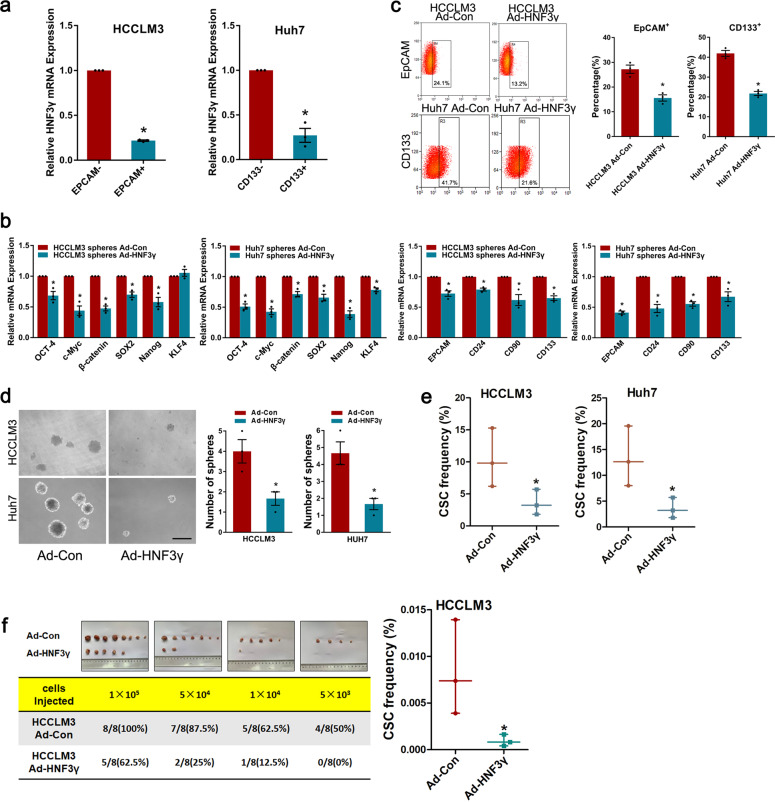
HNF3γ promotes the differentiation of liver CSCs. **a** The expression of HNF3γ in EpCAM^+^ or CD133^+^ HCC cells was determined by real-time PCR. **b** The expression of stemness-associated transcription factors and liver CSC markers in HCCLM3 or Huh7 cells-formed spheres infected with Ad-HNF3γ were analyzed by real-time PCR. **c** Flow cytometry assay of the EpCAM^+^ or CD133^+^ cell populations in HCCLM3 or Huh7 cells infected with Ad-HNF3γ. Results were expressed as means ± SEM. **d** Representative images of spheroids generated from Ad-HNF3γ- or Ad-Con-infected HCC cells. The number of hepatoma spheroids was counted and compared. Results were expressed as means ± SEM. Scale bar, 150 μm. **e**, **f** In vitro and in vivo LDA assay of HCC cells infected with Ad-HNF3γ or Ad-Con. The CSC frequency was calculated as described in “Methods”

Considering that CSCs can differentiate into cancer cells under certain pro-differentiation conditions, we investigated whether HNF3γ could induce the differentiation of liver CSCs. As shown in Fig. 5a, decreased HNF3γ expression was observed in EpCAM^+^ or CD133^+^ HCC cells compared with EpCAM^−^ or CD133^−^ cells, suggesting that HNF3γ expression was further reduced in liver CSCs. Enforced HNF3γ expression suppressed the expression of stemness-associated factors and liver CSC markers in HCC spheres (Fig. 5b) as well as HCC cells (Supplementary Fig. S6a, b). The proportion of EpCAM^+^ or CD133^+^ liver CSCs was significantly decreased in HCC cells infected with Ad-HNF3γ, and Ad-HNF3γ-infected cells formed fewer and smaller spheres compared with control cells, suggesting that HNF3γ could reduce the self-renewal capacity of liver CSCs (Fig. 5c, d and Supplementary Fig. S6c). Most importantly, enforced HNF3γ expression remarkably reduced the CSC frequency both in vitro and in vivo (Fig. 5e, f), which further indicates that HNF3γ can attenuate HCC cells’ stem-like characteristics.

### HNF3γ is a potential target in HCC differentiation therapy

**Fig. 6:**
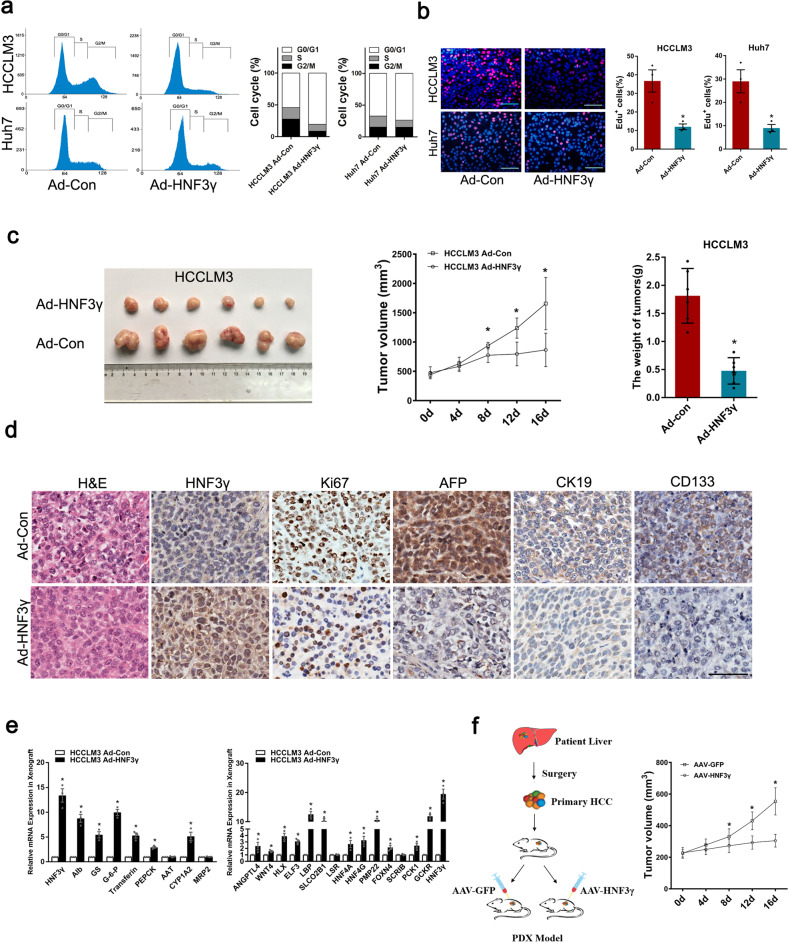
HNF3γ inhibits HCC cell proliferation and xenografted HCC growth. **a** Flow cytometry analysis of HCCLM3 or Huh7 cells infected with Ad-HNF3γ or Ad-Con. **b** Representative images of proliferating HCCLM3 or Huh7 cells indicated by EdU staining are shown. The nuclei were counterstained with DAPI. EdU+ cells were stained with red immunofluorescence. Scale bars, 25 μm. Results were expressed as means ± SEM. **c** HCCLM3-xenografted tumor morphology, tumor growth curve, and average tumor weight in Ad-HNF3γ and Ad-Con groups are shown. **d** Representative images of IHC staining of HNF3γ, Ki67, AFP, CK19, and CD133 in the xenografted tumors are shown. Scale bar, 100 μm. **e** The expression of hepatocyte-specific genes and differentiation-associated genes in HCCLM3-xenografted tumors were examined by real-time PCR. **f** Patient-derived HCC xenografts were treated with either AAV-HNF3γ or AAV-GFP. The size of xenografted tumors was measured every week, and the volume was calculated

To explore the potential value of HNF3γ in HCC differentiation therapy, we investigated whether HNF3γ-mediated differentiation could repress HCC growth. As shown in Supplementary Fig. S7a, b, HNF3γ delivery dramatically inhibited the proliferation and colony formation of HCC cells. Accordingly, the cell cycle analysis revealed a decreased proportion of cells in S phase in Ad-HNF3γ-infected cells (Fig. 6a). In addition, an EdU incorporation assay confirmed the attenuated proliferation of HCC cells infected with Ad-HNF3γ (Fig. 6b). Consistently, HNF3γ delivery dramatically repressed the xenografted HCC growth in vivo (Fig. 6c). IHC staining of Ki67 further confirmed the reduction in cell proliferation in Ad-HNF3γ-treated HCC xenografts (Fig. 6d). As expected, Ad-HNF3γ-administered HCC xenografts exhibited a more differentiated phenotype with suppressed AFP, CK19, and CD133 expression and enhanced expression of hepatocyte-specific genes and hepatic differentiation-related molecules (Fig. 6d, e). Considering the distinguished advantage of adeno-associated virus (AAV) in gene therapy, we further tested the therapeutic effects of AAV-HNF3γ using patient-derived xenograft (PDX) models, and the results showed that AAV-mediated HNF3γ delivery significantly inhibited the HCC progression (Fig. 6f), suggesting that HNF3γ could be an optimized target in HCC differentiation therapy.

### HNF3γ sensitizes HCC cells to sorafenib treatment

**Fig. 7:**
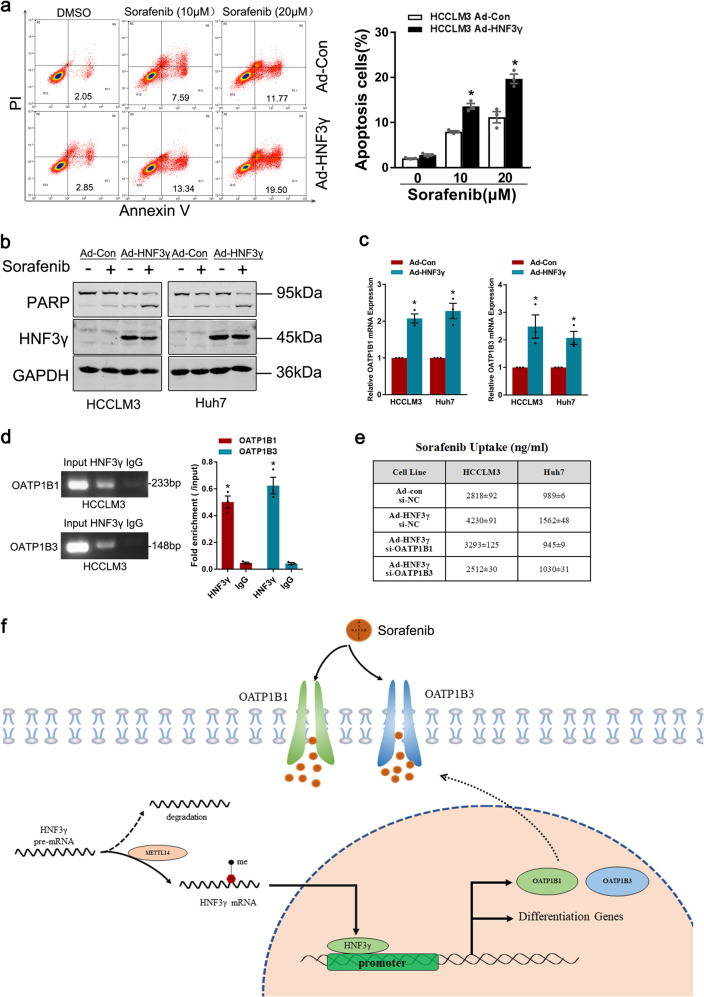
HNF3γ sensitizes HCC cells to sorafenib treatment. **a** HCCLM3 cells infected with Ad-HNF3γ or Ad-Con were treated with sorafenib (10 µM or 20 µM) for 24 h, and apoptotic cells were detected by flow cytometry. Results were expressed as means ± SEM. **b** HCC cells infected with Ad-HNF3γ or Ad-Con were treated with sorafenib (10 µM) for 48 h and then subjected to western blot analysis. **c** The expression of OATP1B1 and OATP1B3 in HCC cells infected with Ad-HNF3γ or Ad-Con was determined by real-time PCR. **d** HCCLM3 cells were subjected to a ChIP assay with anti-HNF3γ or anti-IgG antibody followed by qPCR analysis. **e** HCCLM3 or Huh7 cells were infected with Ad-HNF3γ or Ad-Con followed by siRNA transfection as indicated. The cells were then treated with sorafenib for 48 h, and the intracellular sorafenib was determined by mass spectroscopy as described in “Methods”. **f** Schematic diagram of HNF3γ-enhanced HCC cell differentiation and sorafenib response

Sorafenib has been approved for treating advanced HCC for 12 years, but to date, no biomarker has been used in the clinic to predict its response. Herein, we evaluated the significance of HNF3γ expression in the response of HCC cells and patients to sorafenib. As shown in Fig. 7a, b and Supplementary Fig. S8a, flow cytometry assay and western blot analysis both demonstrated that HNF3γ overexpression notably increased the sorafenib-induced cell apoptosis, suggesting that HNF3γ may enhance the sorafenib response of HCC cells. As expected, interference of METTL14 decreased the apoptosis in HCC cells upon sorafenib treatment (Supplementary Fig. S8b). In the mechanistic study, we first examined the expression levels of sorafenib targets in HCC cells overexpressing HNF3γ, but no alteration in the levels of these targets was found (Supplementary Fig. S8c). Considering that an enhanced drug response might also be due to an increase in drug uptake, we then determined the levels of OATP1B1 and OATP1B3, which are sorafenib influx transporters and primarily responsible for sorafenib uptake in cells. Surprisingly, a remarkable induction of OATP1B1 and OATP1B3 was detected in Ad-HNF3γ-infected HCC cells (Fig. 7c), and a ChIP assay revealed the direct interaction of HNF3γ with the OATP1B1 and OATP1B3 promoter (Fig. 7d). Moreover, interference of OATP1B1 and OATP1B3 suppressed the HNF3γ-induced increase in sorafenib uptake (Fig. 7e), suggesting that HNF3γ might transactivate the expression of sorafenib influx transporters to enhance the sorafenib response in HCC cells (Fig. 7f).

### HNF3γ expression correlates with patient benefit from sorafenib therapy

**Fig. 8:**
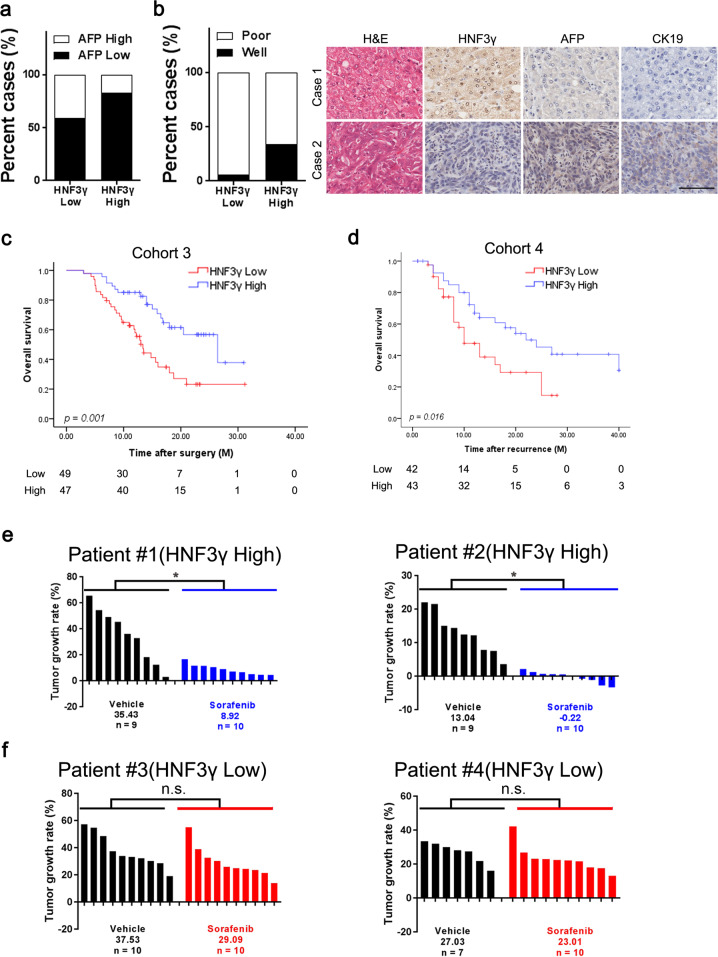
HNF3γ levels in patient HCCs correlate with sorafenib benefit. **a** The correlation between HNF3γ expression and AFP levels was evaluated in 67 HCC patients using a chi-square test. **b** The correlation between HNF3γ expression and HCC differentiation was evaluated in patient HCC tissues using a chi-square test (left). H&E and IHC staining for the dedifferentiation markers AFP and CK19 were conducted, and the representative images are shown (right). Scale bar, 100 μm. **c** HNF3γ expression was examined by IHC staining in HCC samples from 96 patients who had received adjuvant sorafenib therapy after surgery. Overall survival of patients between the HNF3γ-high and HNF3γ-low groups was compared with Kaplan–Meier analysis. **d** HNF3γ expression was examined by IHC staining in primary HCC samples from 85 patients who had received sorafenib treatment for recurrent tumors. The overall survival of patients between the HNF3γ-high and HNF3γ-low groups was analyzed with Kaplan–Meier analysis. **e**, **f** PDXs with high or low HNF3γ expression in their primary HCC tumors indicated by IHC staining scores (details can be found in “Materials and methods”) were treated with sorafenib (30 mg/kg body weight) or vehicle for 28 days. Xenografted tumor growth was monitored, and the tumor growth rates were calculated

We then analyzed the relationship between HNF3γ expression and HCC differentiation in a patient cohort (Supplementary Table S4). Chi-square analysis of HNF3γ levels and clinicopathological characteristics showed that low HNF3γ levels in HCC were correlated with high AFP levels in patient (Fig. 8a and Supplementary Table S4). As expected, low HNF3γ expression was associated with the poor differentiation status of HCC (Fig. 8b and Supplementary Table S4), which further supports a correlation between HNF3γ reduction and HCC dedifferentiation. In further study, the expression of HNF3γ was examined in a cohort of HCC patients who had received adjuvant sorafenib treatment (Supplementary Table S5). Kaplan–Meier analysis revealed that patients with high HNF3γ levels in the resected HCCs exhibited a better OS after adjuvant sorafenib treatment than the patients with low HCC HNF3γ levels (Fig. 8c). We also analyzed HNF3γ levels in primary HCC tissues from another cohort of patients who had been treated with sorafenib after tumor recurrence (Supplementary Table S6). The results showed that high HNF3γ levels were associated with superior patient survival (Fig. 8d). Moreover, PDXs with high HNF3γ levels displayed significant growth inhibition upon sorafenib treatment (Fig. 8e), while PDXs with low HNF3γ expression did not (Fig. 8f). These data suggest that HNF3γ expression is correlated with sorafenib response and could be a novel predictor of sorafenib benefit for HCC patients.

## Discussion

Hepatocarcinogenesis has been regarded as a multistage process involving various genetic and environmental factors.^21^ Interaction and cross-regulation of distinct factors synergistically promote HCC occurrence and contribute to its high heterogeneity.^22^ The extreme heterogeneity of HCC results in its resistance to existing therapies, including targeted treatment. Here, we propose that HNF3γ is evidently downregulated in patient HCC tissues via METTL14-dependent m6A RNA methylation machinery. Reduced HNF3γ expression was correlated with the malignant characteristics of HCC and poor survival of patients. HNF3γ delivery promoted the differentiation of HCC cells as well as liver CSCs, which led to the suppression of HCC growth and the enhancement of sorafenib response. Moreover, HNF3γ transactivated the expression of sorafenib influx transporters OATP1B1 and OATP1B3 to enhance the sorafenib response of HCC cells.

In the present study, we first reported the reduction of HNF3γ in human HCC and observed its correlation with patient survival, which aroused our interest to delineate the mechanism underlying its downregulation in HCC. DNA methylation and histone acetylation play important roles in regulation of numerous genes.^23,24^ However, we excluded their influence on the HNF3γ reduction in HCC cells using specific inhibitors. The interference of Dicer did not affect HNF3γ expression, ruling out the effect of miRNA on HNF3γ downregulation. Emerging studies have demonstrated that m6A modification of mRNAs plays a critical role in RNA fate, including mRNA stability, splicing, transport, localization, and translation.^25,26^ Methylation of adenosine at the N6 position is mediated by a multicomponent complex composed of METTL3, METTL14, and WTAP in mammals.^17,27^ Demethylation of m6A has been reported to be modulated by FTO and ALKBH5 to date.^28^ In the present study, we propose that HNF3γ expression is regulated by RNA methylation based on the observation that interference of m6A methylase resulted in a decrease of HNF3γ and knockdown of m6A demethylase led to an increase of HNF3γ. Furthermore, we found that METTL14 but not other m6A methylases or demethylases could be involved in the HNF3γ reduction in patient HCCs. In addition, we identified that IGF2BPs were involved in the stabilization of m6A-modified HNF3γ mRNA. The molecular mechanism underlying IGF2BPs-enhanced RNA stability has been reported by Huang et al.^18^ The naive mRNA with m6A modifications was preferentially recognized by IGF2BP proteins. By recruiting mRNA stabilizers, such as HuR and MATR3, IGF2BPs protect target mRNA from degradation in the P-body.^18^ Given that the importance of HNFs in liver development and hepatocarcinogenesis has been widely accepted, the molecular mechanism underlying the reduction of HNFs in human disease remains obscure thus far. To the best of our knowledge, this is the first study to report the involvement of m6A modification in HNF dysregulation in cancer. Whether m6A modification is required for the reduction of other HNFs in HCC is worthy of investigation.

HNF3γ has been reported to play an essential role in hepatic metabolism regulation in mature hepatocytes,^29^ but its role in HCC differentiation remains unreported. Here, we observed the induction of HNF3γ during hepatic differentiation and its reduction in the dedifferentiated HCC cells. Consistently, we found that HNF3γ was highly expressed in adult liver tissues but lowly expressed in fetal liver or liver cancer, suggesting a role of HNF3γ in the maintenance of hepatocyte differentiation status. In further study, delivery of HNF3γ into HCC cells increased the expression of hepatocyte-specific biomarkers and enhanced hepatic functions, suggesting a promotive role of HNF3γ in HCC cell differentiation. Accumulating evidence has shown that CSCs in solid tumors are responsible for cancer relapse, metastasis, and chemoresistance.^30^ It has been reported that normal or neoplastic non-stem cells can spontaneously convert to a stem-like state.^31^ Recent studies have also suggested that non-stem cancer cells can be converted to CSCs following a dedifferentiation process.^32,33^ We previously reported that acquisition of stemness after transformation of liver progenitor cells gives rise to liver CSCs.^34,35^ Interestingly, enforced expression of HNF3γ dramatically reduced CSC frequency and suppressed CSC expansion, demonstrating that HNF3γ can induce the differentiation of liver CSCs.

Maintenance of epithelial morphology and hepatic function is cross-regulated by a few liver-enriched transcription factors, in particular hepatocyte nuclear factors.^36^ HNF3γ is expressed at high levels in the mature liver and is predicted to bind the promoter of numerous genes in hepatocytes.^37^ Our RNA-seq and bioinformatics analysis suggested an HNF3γ-centered regulatory network consisting of the liver differentiation-related transcription factors. These transcription factors and functional molecules could synergistically regulate the expression of numerous hepato-specific genes, thus promoting the differentiation of HCC cells and liver CSCs. As expected, we found that HNF3γ-mediated HCC cell differentiation led to the inhibition of HCC cell proliferation in vitro and suppression of xenografted HCC growth in vivo. To our knowledge, this is the first report concerning the role of HNF3γ in HCC differentiation, and our results suggest the clinical significance of HNF3γ in HCC differentiation therapy.

Sorafenib, a multikinase inhibitor, is the first FDA-approved targeted agent for advanced HCC, but it only exhibits a notable therapeutic effect on a minority of patients.^38^ Therefore, identifying novel biomarkers for patient selection to improve sorafenib efficacy remains a primarily unmet need. It has been reported that the differentiation status of cancer is associated with its response to targeted therapies, but the underlying molecular mechanism remains obscure.^39^ Our data showed that enforced HNF3γ expression sensitized HCC cells to sorafenib-induced growth inhibition and cell apoptosis. The mechanistic study on the synergistic effect of HNF3γ and sorafenib in HCC cell apoptosis demonstrated that HNF3γ upregulated OATP1B1 and OATP1B3 expression and thus enhanced sorafenib uptake, resulting in the enhanced sensitivity of HCC cells to sorafenib administration. These findings provide a new explanation for the enhanced response of well-differentiated tumors to chemotherapeutics. Consistently, our clinical investigation revealed that patients with high HCC HNF3γ levels benefited from sorafenib therapy but patients with low HNF3γ levels did not. Since the histology and gene expression patterns of PDX model were highly consistent between xenografts and case-matched original tumors,^40^ we further validate the correlation between HNF3γ expression and sorafenib response using PDX model. Our results showed that PDXs with high HNF3γ levels displayed significant growth inhibition upon sorafenib treatment, while PDXs with low HNF3γ expression did not respond to sorafenib. Collectively, these findings suggest that HNF3γ may serve as a predictor of sorafenib benefit in HCC personalized therapy, which warrants further investigation.

## Materials and methods

### Patients and HCC samples

A total of 580 HCC and corresponding paracancerous tissues were obtained from patients that underwent surgical resection from 2006 to 2010 in Eastern Hepatobiliary Surgery Hospital (EHBH). The specimen collection procedure was approved by the Ethics Committee of EHBH. A tissue microarray containing 156 HCCs from patients with follow-up information (Cohort 1) was used for survival analysis. The differentiation status of HCC tissues from 67 patients in Cohort 2 was evaluated blindly by a pathologist. A total of 96 primary HCC patients that received adjuvant sorafenib therapy after surgery at EHBH from 2009 to 2011 were included in Cohort 3 and followed for 36 months. A total of 85 patients who received sorafenib for the recurrent tumors at EHBH from 2008 to 2015 were included in Cohort 4. Detailed clinicopathological information is provided in Supplementary Tables 1, 2, 5 and 6. OS and recurrence-free survival (DFS) were analyzed using the Kaplan–Meier method as described previously.^41^ OS was defined as the interval between the date of surgery and death. DFS was defined as the interval between the date of surgery and recurrence. If recurrence was not diagnosed, cases were censored on the date of the last follow-up. Sixty-three paired cDNA samples from HCC and corresponding para-carcinoma tissues from patients with follow-up information were used for survival analysis. Tissue lysates of 18 paired HCC and para-carcinoma tissues from patients were used for western blot analysis of HNF3γ expression. Seventeen peri-tumoral normal liver tissues, paired HCC samples, and PVTT were used for real-time PCR analysis. Twenty-one HCC samples with paired recurrent foci were diagnosed by two independent pathologists. In addition, a set of 57 fresh patient HCC tissues were used for analyzing the correlation between HNF3γ mRNA and m6A modification-related molecules.

### Cell lines and adenoviruses

The HCC cell lines HCCLM3 (p53 wide type) and Huh7 (p53 mutant) were obtained from Cell Bank of Type Culture Collection of Chinese Academy of Sciences.^42^ Serum-Free Cell Freezing Medium was brought from New Cell & Molecular Biotech. Adenoviruses expressing GFP or HNF3γ (designated as Ad-Con and Ad-HNF3γ) were purchased from Viagen (Shandong, China). The sequences of small interfering RNA are listed in Supplementary Table S7.

### Hepatic function assays

To determine the secretion of human albumin and urea, HCC cells were seeded in 6-well plates for 24 h, and the medium was then replaced with fresh DMEM without fetal bovine serum for another 24 h. The supernatant was collected according to the manufacturer’s instructions. Human albumin was measured with a Human Albumin ELISA Quantitation kit (Assay Pro), and urea was measured with a QuantiChrom^TM^ Urea Assay Kit (BioAssay System). The Periodic-Acid Schiff staining kit was obtained from Sigma-Aldrich. Cells were fixed and stained following the manufacturer’s instructions. For oil red O staining, HCC cells were stained with oil red O (Sigma-Aldrich) according to the manufacturer’s instructions.

### In vitro cell behavior assay

For cell proliferation assays, 3 × 10^3^ HCC cells were seeded into each well of 96-well plates, and then, ATP activity was measured using Cell Counting Kit-8 (Dojindo, Kumamoto, Japan) to assess cell proliferation. 5-Ethynyl-2-deoxyuridine (EdU) incorporation assays were performed using an EdU Kit (RiboBio) according to the manufacturer’s instructions. For anchor-independent growth assays, HCC cells (2 × 10^3^ cells/ml) were mixed with Matrigel Basement Membrane Matrix (BD Bioscience, Bedford, MA) at a ratio of 2:1 to a final volume of 150 µl and then cultured in 96-well plates for 7 days. Colonies formed within the gel were counted, and representative pictures were taken.

### Real-time PCR and western blotting

Total RNA was isolated from tissues or cells using Trizol (Invitrogen). cDNA synthesis was performed using a Reverse Transcription System (Promega). The original amount of the specific transcripts was analyzed via real-time PCR using a Roche Light Cycler 96 System (Roche, USA) and a SYBR Green PCR Kit (Roche). The primer sequences are listed in Supplementary Table 7. Relative mRNA expression levels were calculated by the 2^−^^ΔΔCt^ method. All results of relative expression values were normalized to β-actin, and shown as the mean ± SEM of triplicate experiments. Cell lysate or human HCC extract samples were analyzed by immunoblot using primary antibodies and IRDye 800CW-conjugated secondary antibody (LI-COR Biosciences) as previously described.^42^ The antibodies used are listed in Supplementary Table S8.

### Immunohistochemical analysis

Immunohistochemistry or H&E staining were performed as described previously.^42^ The primary antibodies used are listed in Supplementary Table 8. HNF3γ expression was scored through microscope and the score was defined as “0” (negative), “0.5” (positivity ≤ 20%), “1” (20% < positivity ≤ 35%) “1.5” (35% < positivity ≤ 50%), “2” (50% < positivity ≤ 65%), “2.5” (65% < positivity ≤ 80%), “3” (80% < positivity ≤ 90%), or “3.5” (90% < positivity ≤ 100%). Staining scores <2 in HCC samples were defined as low expression. Staining ≥2 in HCC samples were defined as high expression.

### m6A meRIP-qPCR

MeRIP assays were performed following the manufacturer’s instructions (Magna MeRIP m6A Kit). Briefly, RNA from HCC cells or HCC tissues was isolated and fragmented by sonication for 10 s at 0 °C. Magnetic beads (MedChemExpress) were incubated with anti-m6A antibody for 30 min at room temperature. Then, the anti-m6A antibody-bound magnetic beads were washed, added to DNA-free RNA, and incubated for 2 h at 4 °C with rotation. The beads were then washed and the bound RNA was eluted using 5′-monophosphate sodium salt (m6A). The eluted RNA was purified with a miRNeasy mini kit (QIAGEN) and then subjected to qPCR (real-time PCR).^43^

### Limiting dilution assay

For in vitro limiting dilution assays (LDAs), HCCLM3, or Huh7 cells were seeded into 96-well ultra-low attachment culture plates at serially diluted cell numbers and incubated for 7 days. CSC proportions were analyzed using Poisson distribution statistics and the L-Calc Version 1.1 software program (Stem Cell Technologies, Inc., Vancouver, Canada). For in vivo LDAs, HCCLM3 cells were serially diluted to the desired doses and then injected subcutaneously into NOD-SCID mice. The number of tumors was determined after 2 months, and the CSC proportion was analyzed using ELDA software (http://bioinf.wehi.edu.au/software/elda/index.html) provided by the Walter and Eliza Hall Institute.^42^

### Sorafenib uptake assay

HCCLM3 or Huh7 cells infected with Ad-Con or Ad-HNF3γ were seeded into 6-well plates and were then transfected with si-OATP1B1 or si-OATP1B3. After 12 h cultivation, all cells were treated with sorafenib at a concentration of 10 μmol/l. The culture media was replaced after 48 h and the cells were rapidly rinsed by gently dispensing 10 ml deionized water. Then cells in each well were quenched by directly adding 1 ml of MeOH (−80 °C), then transferred to a −80 °C freezer for 20 min. After 20 min, cells were scraped to 1.5 ml microcentrifuge tubes and suspended for 1 min. Extracts were centrifuged at 4 °C for 3 min at 16,100 × *g*. Sorafenib in the supernatants was analyzed by LC-MS/MS (Agilent 1290 Infinity II liquid chromatograph) as described previously.^44^

### Experimental animal models

For in vivo tumor growth assays, HCCLM3 cells were subcutaneously injected at 2 × 10^6^ cells per mouse into nude mice. When xenografted tumor growth reached 500 mm^3^, the mice were subjected to intratumoral injection of Ad-con or Ad-HNF3γ every other day. Xenografted tumor growth was monitored as described previously.^43^ For the PDX model, fresh patient HCC tissues were cut into fragments with a volume of 3 × 3 mm^3^ and then implanted subcutaneously into the flanks of nude mice. The mice were given sorafenib (30 mg/kg) or vehicle orally twice a week for 24 days. Tumor growth was measured at the indicated time points. This procedure was approved by the Ethics Committee of EHBH.

### Statistical analysis

Statistical analysis was performed using SPSS 16.0 software (SPSS Inc., USA). The data were expressed as the mean ± SEM. The significance of mean values between two groups was analyzed via Mann–Whitney *U* test or Student’s *t* test. Pearson’s correlation analysis was performed to test the correlation between two variables. *p* values <0.05 were considered statistically significant.

## Supplementary information

Supplementary materials

## Data Availability

All data that support the findings of this study are available to the researchers on reasonable request.
