# Supplementary material for: m6A RNA methylation-mediated HNF3γ reduction renders hepatocellular carcinoma dedifferentiation and sorafenib resistance
Source: Signal Transduct Target Ther. 2020 Dec 26;5:296. doi: 10.1038/s41392-020-00299-0 (PMC7762754; doi:10.1038/s41392-020-00299-0)
Supplement: Supplementary file 1 — Supplementary materials [file 41392_2020_299_MOESM1_ESM.doc]

Supplementary Materials for

**m6A RNA methylation-mediated HNF3γ reduction renders hepatocellular carcinoma dedifferentiation and sorafenib resistance**

Tengfei Zhou^1, #^, Shichao Li^1, #^, Daimin Xiang^1, #^, Junyu Liu^1^, Wen Sun^1^, Xiuliang Cui^1^, Beifang Ning^2^, Xiao Li^3^, Zhuo Cheng^1^, Weiqi Jiang^1^, Cheng Zhang^1^, Xijun Liang^1^, Liang Li^1^, Xin Cheng^3^, Liu Hui^4,*^, Hongyang Wang^1,5,*^ and Jin Ding^1,5,6,*^

*^1^International Cooperation Laboratory on Signal Transduction, Eastern Hepatobiliary Surgery Hospital/Institute, the Second Military Medical University, Shanghai, China.*

*^2^Department of Gastroenterology, Changzheng Hospital, the Second Military Medical University, Shanghai, China;*

*^3^Shanghai Institutes for Biological Sciences, Chinese Academy for Sciences, China.*

^4^*The Third Department of Hepatic Surgery, Eastern Hepatobiliary Surgery Hospital, the Second Military Medical University, Shanghai, China.*

*^5^National Center for Liver Cancer, Shanghai, China.*

*^6^Tongji University School of Medicine, Shanghai, China.*

*^#^These authors contributed equally to this work.*

**Corresponding authors.*

**Corresponding Authors:** Dr. Jin Ding (dingjin1103@163.com) and Dr. Hongyang Wang (hywangk@vip.sina.com). International Cooperation Laboratory on Signal Transduction, Eastern Hepatobiliary Surgery Institute, Second Military Medical University, 225 Changhai Road, 200438 Shanghai, China. Dr. Hui Liu (liuhuigg@hotmail.com). The Third Department of Hepatic Surgery, Eastern Hepatobiliary Surgery Hospital, the Second Military Medical University, 225 Changhai Road, 200438 Shanghai, China.

**This file includes:**

Supplementary Materials and Methods

Supplementary Figures. S1 to S8

Supplementary Tables S1 to S8

**Supplementary Materials and Methods**

**Chromatin immunoprecipitation (ChIP) assay**

ChIP assays were performed using EpiTect ChIP qPCR Kit (QIAGEN) following the manufacturer’s instructions. HCCLM3 cells were crosslinked with 1% formaldehyde at 37°C for 10 minutes and collected into PBS with PMSF. The samples were then subjected to ice bath ultrasound to shear genomic DNA, which break most DNA into 200-1000 bp size. Chromatins were immunoprecipitated with Protein A+G Agarose, anti-IgG and anti-HNF3γ antibodies respectively. Then, the DNA was purified using EDTA, Tris pH 6.5 and glycogen. PCR was performed to examine the bound sequences, and the primers were listed in Supplementary Table 7.

**Analysis of mRNA degradation rate**

To determine the mRNA degradation rate, HNF3γ mRNA levels were assessed in the presence of the transcription inhibitor actinomycin D. HCCLM3 or Huh7 cells were cultured in 12-well plates and transfected with si-NC or si-METTL14. The cells were incubated with actinomycin D (10 μM) for 15 min, 30 min, 60 min, 120 min, 180 min, 240 min or 300 min. Total RNA was extracted by trizol and Real-time PCR was conducted to quantify the relative levels of HNF3γ mRNA. The degradation curves were achieved using GraphPad software.

**Luciferase reporter assay**

DNA fragments of HNF3γ-3'UTR and HNF3γ-CDS containing the wild type m6A motifs as well as mutant motifs (m6A was replaced by T) were directly synthesized and inserted into downstream of firefly luciferase of pMIR-REPORT vector (Obio Technology, Shanghai). For luciferase reporter assay, 100 ng wild-type or mutant HNF3γ-3'UTR (or HNF3γ-CDS) and 20 ng pRL-TK (renilla luciferase control reporter vector) were co-transfected into HCCLM3 cells that had been transfected with si-NC or si-METTL14. The relative luciferase activity of each group in triplicate was analyzed by Dual-Luciferase Reporter Assay Kit (Promega).

(1) HNF3γ-3'UTR with wild-type m6A sites:

CAGGGGTTGG**GAACA**TGGTGGTGGGTATGGCTGGAGCTCACACCACGAAGCTCTTGGGGCCTGATCCTTCTGGTGACACTTCACTTGTCCCATTGGTTAACATCTGGGTGGGTCTATTACTTACTGTGATGACTGCTGTCTCAGTGGGCATGGTGTTGATCCACGGGGTACTGTGATAACCACCATGGATACATTTTGGTGGCCCACTGGGTACTGTGA**GGACT**GCTACATTGATGGATGTTATTGGCTAATCCACTGCATGGTTTGATGGCCACCATCTCGGTTGGCCCTTTGGGTGTGATGGTGATAGCATTTCAGTGACATCTTCTTTGGCCCCCCCCATTAGGTGCTGTGCCCACTTCTTTTTTGGTGTACTTGGCACAGTAGGTGCCAAGTTGGCCACCATTCTGTGTAACACCTTTTTTGGCCCATTGGGTGCTTTGA**TGGACA**TCATACTGGGTAGGTGACAACGTCAGTGGGCCACCATGTGCCATGATGGCTGCTGCAGCCCCGTGTTGGCCATGTCGTCACCATTCTCTCTGGCATGGGTTGGGTAGGGGATGGAGGTGAGAATACTCCTTGGTTTTCTCTGAAGCCCACCCTTTCCCCCAACTCTGGTCCAGGAG**AAACC**AGAAAAGGCTGGTTAGGGTGTGGGGAATTTCTACT

HNF3γ-3'UTR with mutant m6A sites:

CAGGGGTTGG**GATCA**TGGTGGTGGGTATGGCTGGAGCTCACACCACGAAGCTCTTGGGGCCTGATCCTTCTGGTGACACTTCACTTGTCCCATTGGTTAACATCTGGGTGGGTCTATTACTTACTGTGATGACTGCTGTCTCAGTGGGCATGGTGTTGATCCACGGGGTACTGTGATAACCACCATGGATACATTTTGGTGGCCCACTGGGTACTGTGA**GGTCT**GCTACATTGATGGATGTTATTGGCTAATCCACTGCATGGTTTGATGGCCACCATCTCGGTTGGCCCTTTGGGTGTGATGGTGATAGCATTTCAGTGACATCTTCTTTGGCCCCCCCCATTAGGTGCTGTGCCCACTTCTTTTTTGGTGTACTTGGCACAGTAGGTGCCAAGTTGGCCACCATTCTGTGTAACACCTTTTTTGGCCCATTGGGTGCTTTGA**TGGTCA**TCATACTGGGTAGGTGACAACGTCAGTGGGCCACCATGTGCCATGATGGCTGCTGCAGCCCCGTGTTGGCCATGTCGTCACCATTCTCTCTGGCATGGGTTGGGTAGGGGATGGAGGTGAGAATACTCCTTGGTTTTCTCTGAAGCCCACCCTTTCCCCCAACTCTGGTCCAGGAG**AATCC**AGAAAAGGCTGGTTAGGGTGTGGGGAATTTCTACT

(2) HNF3γ-CDS with wild-type m6A sites:

GTGAAAAAAGGGGGCAGCGGGGCTGCCACCACCACCAGGAACG**GGACA**GGGTCTGCTGCCTCGACCACCACCCCCGCGGCCACAGTCACCTCCCCGCCCCAGCCCCCGCCTCCAGCCCCTGAGCCTGAGGCCCAGGGCGGGGAAGATGTGGGGGCTC**TGGACT**GTGGCTCACCCGCTTCCTCCACACCCTATTTCACTGGCCTGGAGCTCCCAGGGGAGCTGAAGC**TGGAC**GCGCCCTACAACTTCAACCACCCTTTCTCCATCAACAACCTAATGTCA**GAACAGA**CACCAGCACCTCCC**AAACTGGAC**GTGGGGTTTGGGGGCTACGGGGCTGAAGGTGGGGAGCCTGGAGTCTACTACCAGGGCCTCTATTCCCGCTCTTTGCTTAATGCATCCTAG

HNF3γ-CDS with mutant m6A sites:

GTGAAAAAAGGGGGCAGCGGGGCTGCCACCACCACCAGGAACG**GGTCA**GGGTCTGCTGCCTCGACCACCACCCCCGCGGCCACAGTCACCTCCCCGCCCCAGCCCCCGCCTCCAGCCCCTGAGCCTGAGGCCCAGGGCGGGGAAGATGTGGGGGCTC**TGGTCT**GTGGCTCACCCGCTTCCTCCACACCCTATTTCACTGGCCTGGAGCTCCCAGGGGAGCTGAAGC**TGGTC**GCGCCCTACAACTTCAACCACCCTTTCTCCATCAACAACCTAATGTCA**GATCAGT**CACCAGCACCTCCC**AATCTGGTC**GTGGGGTTTGGGGGCTACGGGGCTGAAGGTGGGGAGCCTGGAGTCTACTACCAGGGCCTCTATTCCCGCTCTTTGCTTAATGCATCCTAG

**Supplementary Figures**

**
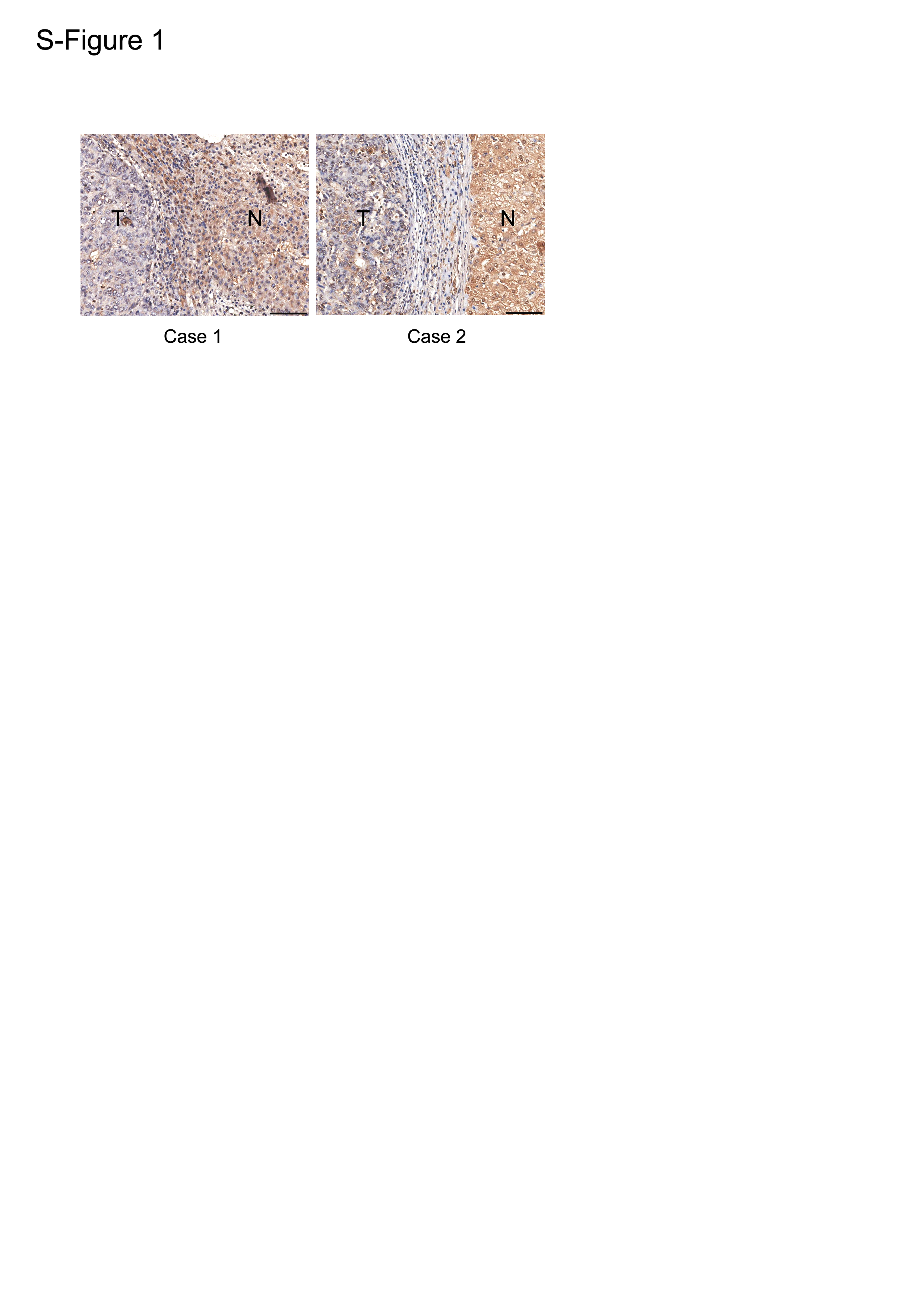
**

**Supplementary Figure 1**

Representative views of HNF3γ expression in patient HCC nodules determined by IHC staining were shown. Scale bar,100μm.

**
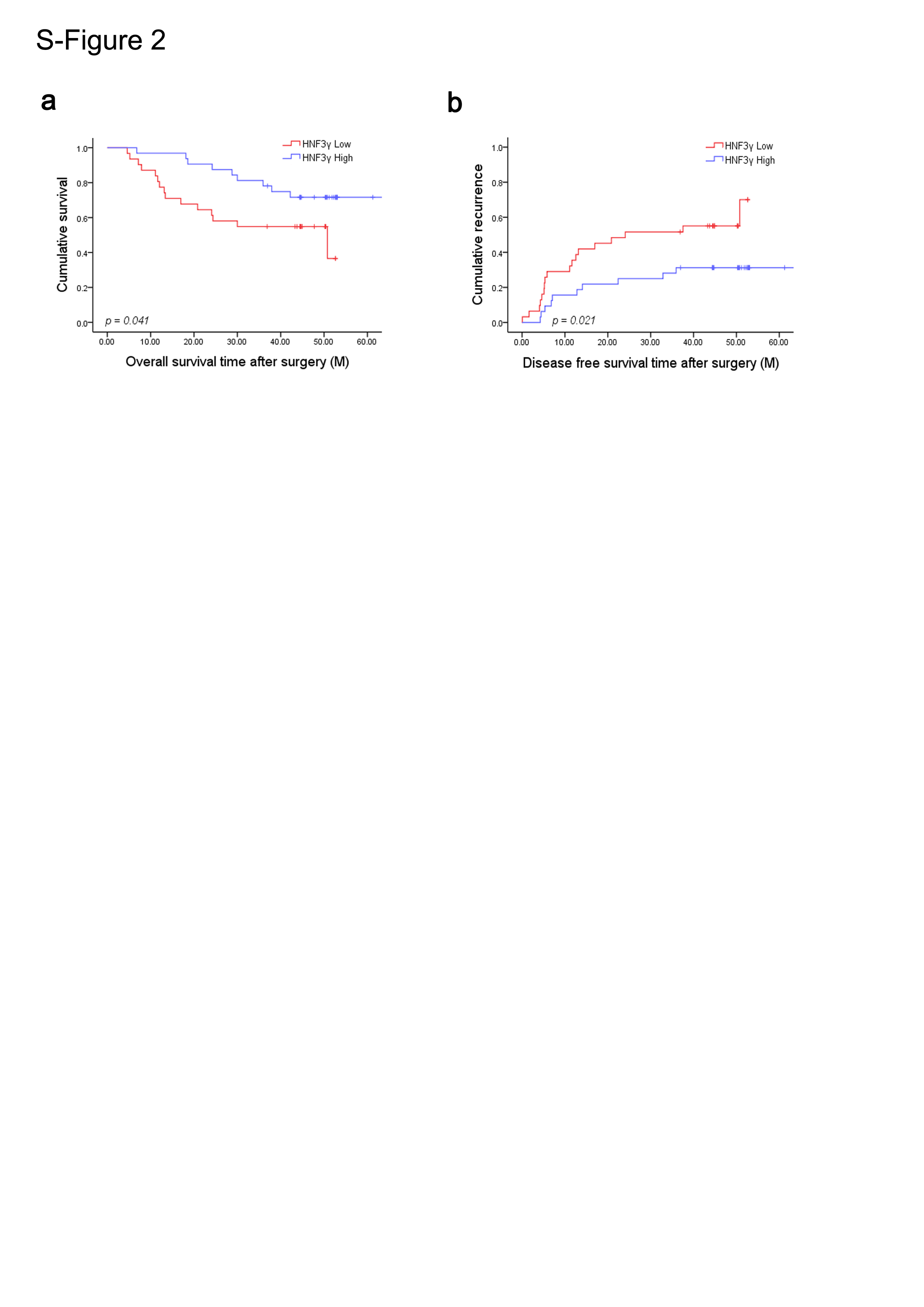
**

**Supplementary Figure 2**

a & b. The overall survival and disease-free survival time after surgery of 63 HCC patients were compared between the ‘‘HNF3γ low’’ (n = 31) and ‘‘HNF3γ high’’ (n = 32) groups using Kaplan-Meier analysis.


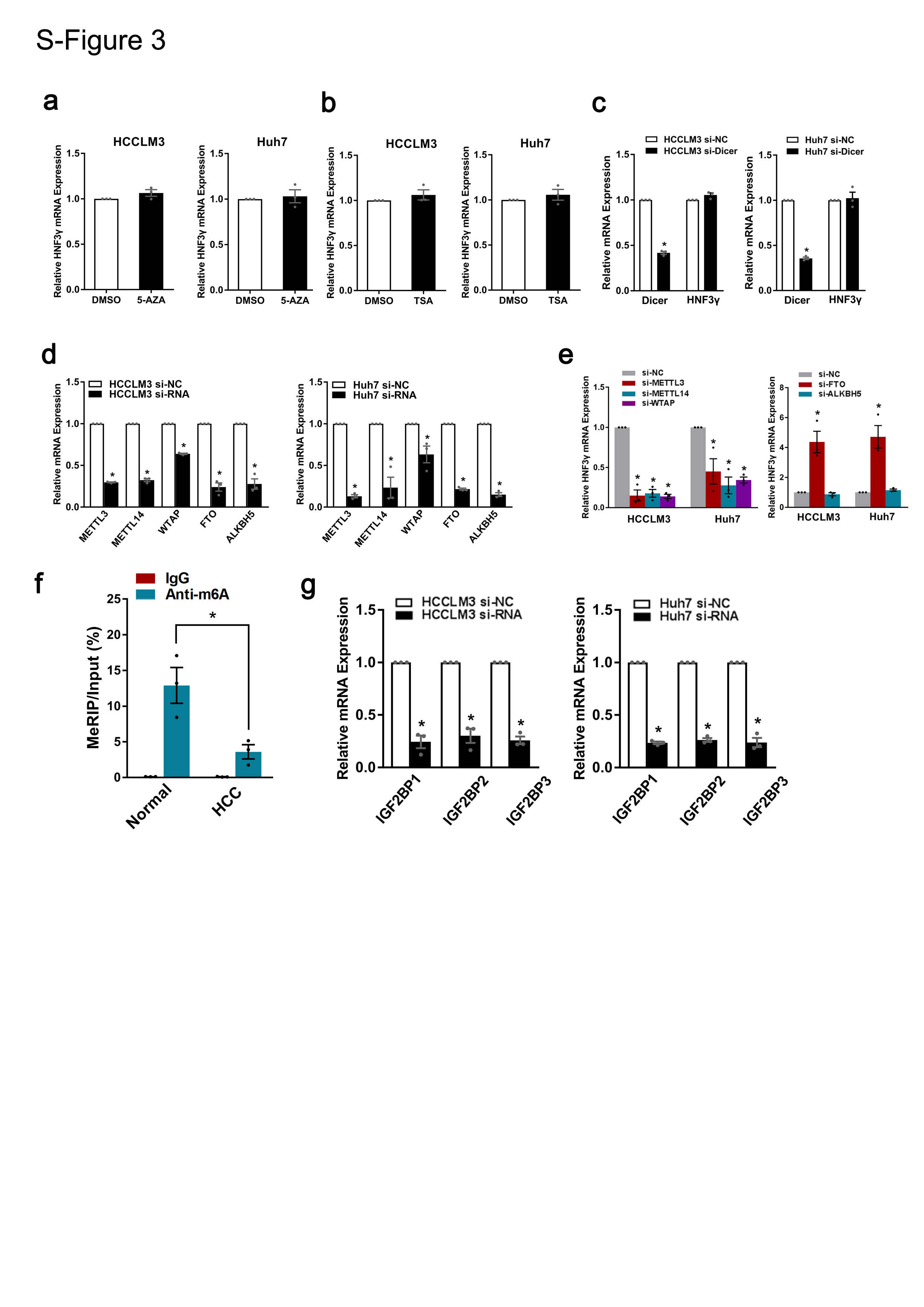


**Supplementary Figure 3**

a. HCCLM3 and Huh7 cells treated with DMSO or 5-AZA as indicated were subjected to real-time PCR analysis.

b. HCCLM3 and Huh7 cells treated with DMSO or TSA as indicated were subjected to real-time PCR analysis.

c. HCCLM3 and Huh7 cells transfected with si-Dicer or si-Control as indicated were subjected to real-time PCR analysis.

d. HCCLM3 and Huh7 cells transfected with the indicated siRNA (si-METTL3, si-METTL14, si-WTAP, si-FTO, si-ALKBH5 or si-Control) were subjected to real-time PCR assay.

e. HCCLM3 and Huh7 cells were transfected with si-METTL3, si-METTL14, si-WTAP, si-FTO, si-ALKBH5 or si-NC as indicated for 48 hours followed by real-time PCR assay (*p < 0.05).

f. Total RNA was isolated from ten patient HCCs and the paracancerous normal tissues respectively using trizol reagent. Equal amount of RNA from ten patient HCCs or ten paracancerous normal tissues were pooled as “HCC” group or “Normal” group respectively as indicated. Me-RIP-qRCR was performed as described in the Materials and methods.

g. HCCLM3 and HUH7 cells transfected with si-IGF2BP1, si-IGF2BP2, si-IGF2BP3 or si-Control as indicated were subjected to real-time PCR analysis.

**
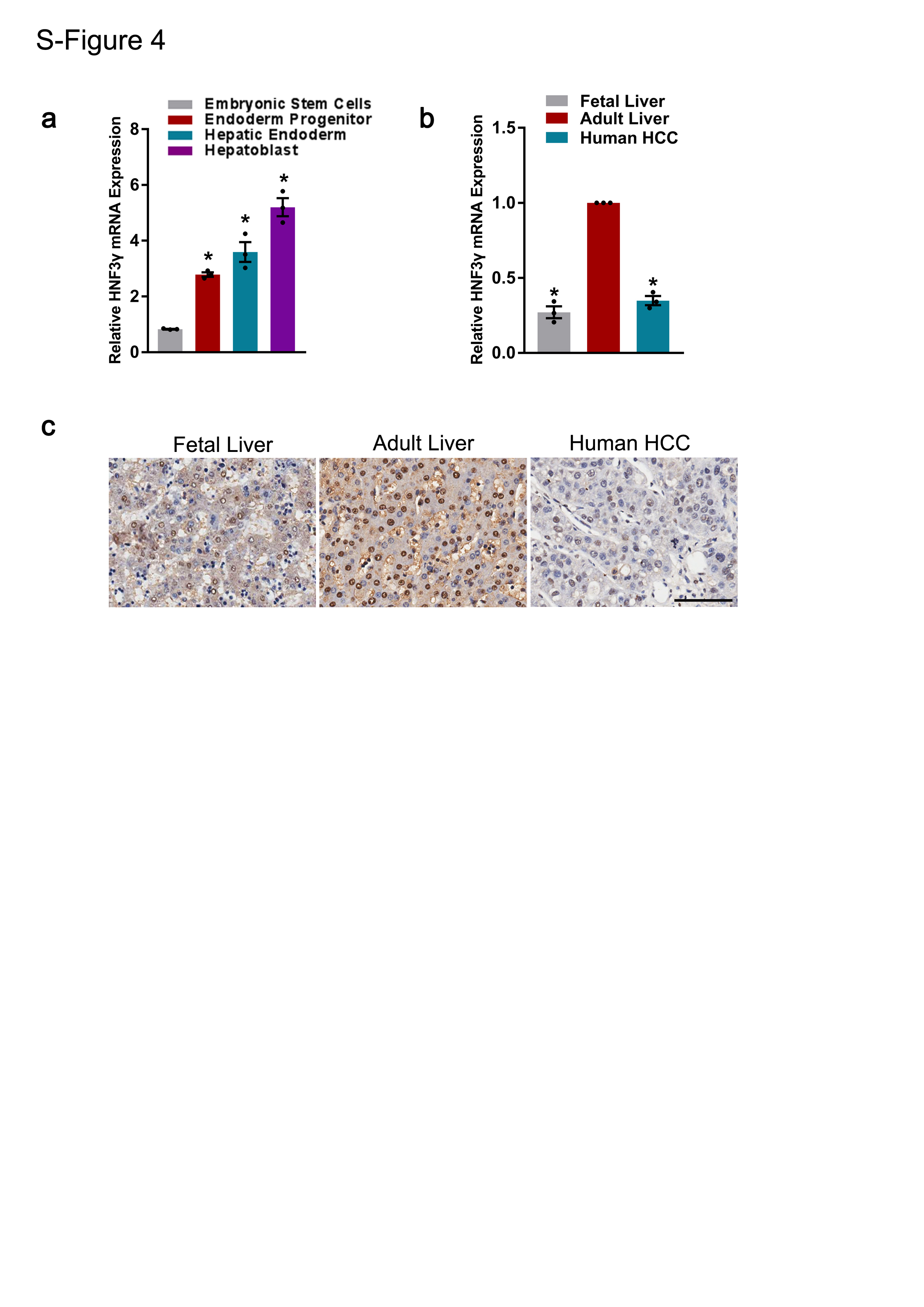
**

**Supplementary Figure 4**

a. HNF3γ expression was investigated during *in vitro* hepatic-specific differentiation by real-time PCR assay.

b. The expression of HNF3γ in fetal liver, adult liver and human HCC was detected by real-time PCR.

c. IHC analysis of HNF3γ expression in human fetal liver, adult liver and HCC tissues. Scale bar,100μm.
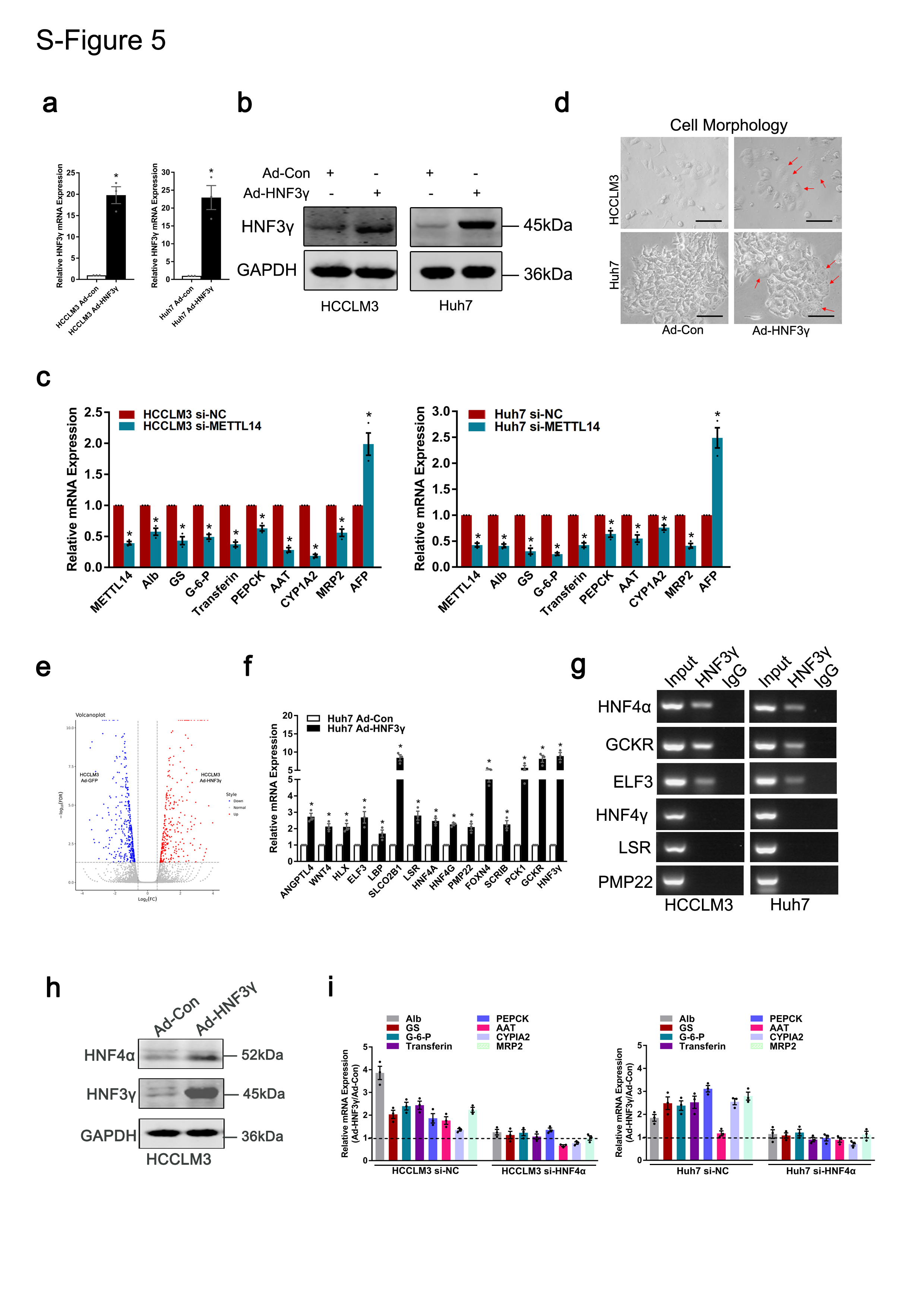


**
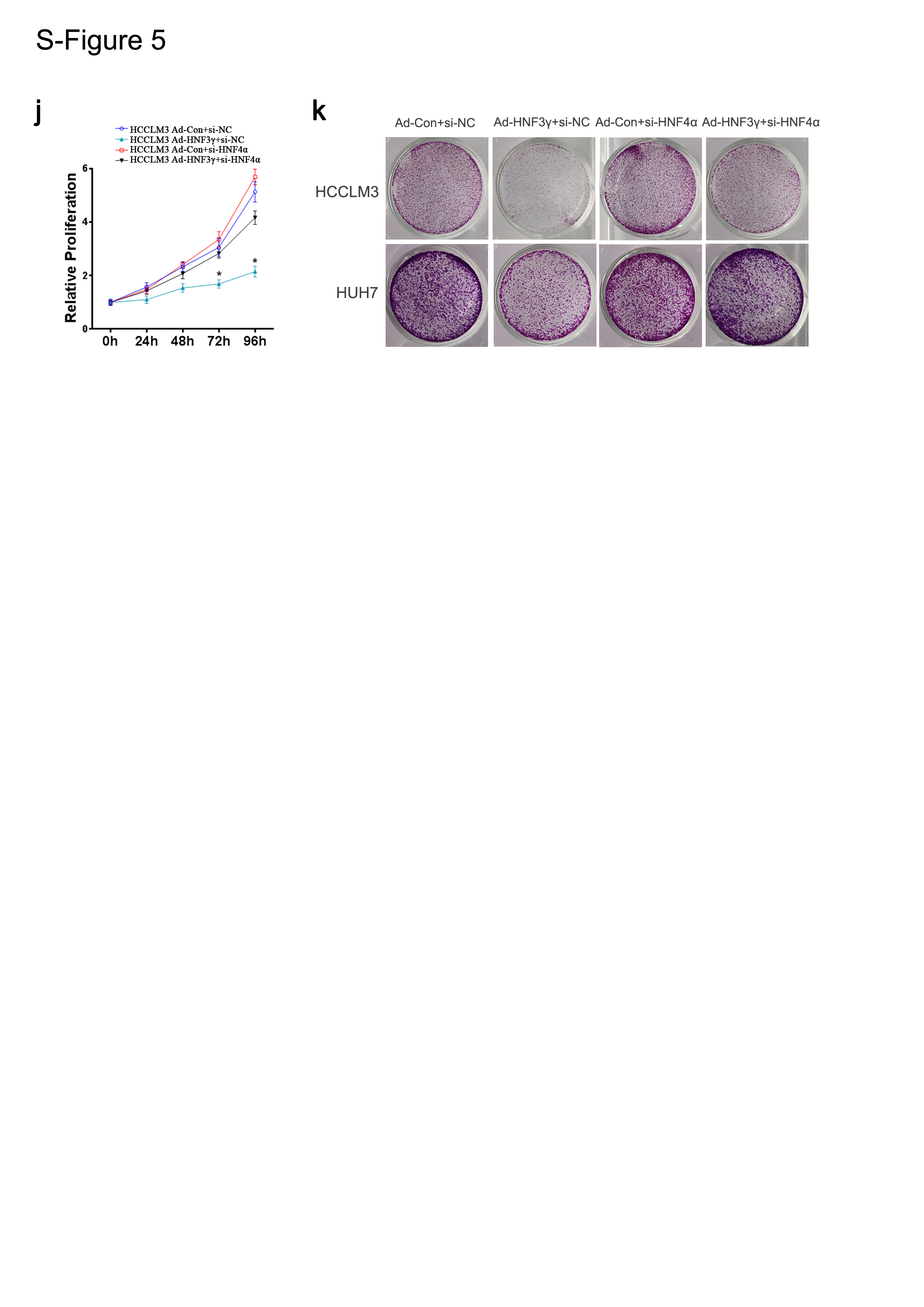
**

**Supplementary Figure 5**

a & b. Overexpression of HNF3γ in HCCLM3 and Huh7 cells was identified by real-time PCR and western blot assay.

c. The expression of hepatocyte-specific genes and AFP in HCCLM3 or Huh7 cells transfected with si-METTL14 or si-NC was determined by real-time PCR.

d. HCCLM3 and Huh7 cells infected with Ad-Con or Ad-HNF3γ were cultured for 5-7 days. The cell morphology was observed through microscopy and the pictures were taken. The red arrows indicate the cells with morphological change.

e. Volcano plot of RNA-sequencing data (HCCLM3 Ad-HNF3γ versus HCCLM3 Ad-Con) is shown.

f. Hepatocyte differentiation-associated genes differentially expressed in HNF3γ-overexpressing Huh7 cells were validated by real-time PCR.

g. HCCLM3 and Huh7 cells were subjected to ChIP assay with anti-HNF3γ or anti-IgG antibody.

h. HCCLM3 cells were infected with Ad-Con or Ad-HNF3γ for 48 hours followed by western blot assay.

i. HCC cells transfected with si-NC or si-HNF4α were infected with Ad-Con or Ad-HNF3γ respectively followed by real-time PCR assay for the expression of hepatocyte-specific genes.

j. HCCLM3 cells transfected with si-NC or si-HNF4α were infected with Ad-Con or Ad-HNF3γ respectively followed by CCK8 assay.

k. HCCLM3 or Huh7 cells transfected with si-NC or si-HNF4α were infected with Ad-Con or Ad-HNF3γ respectively and then subjected to colony growth assay.

**
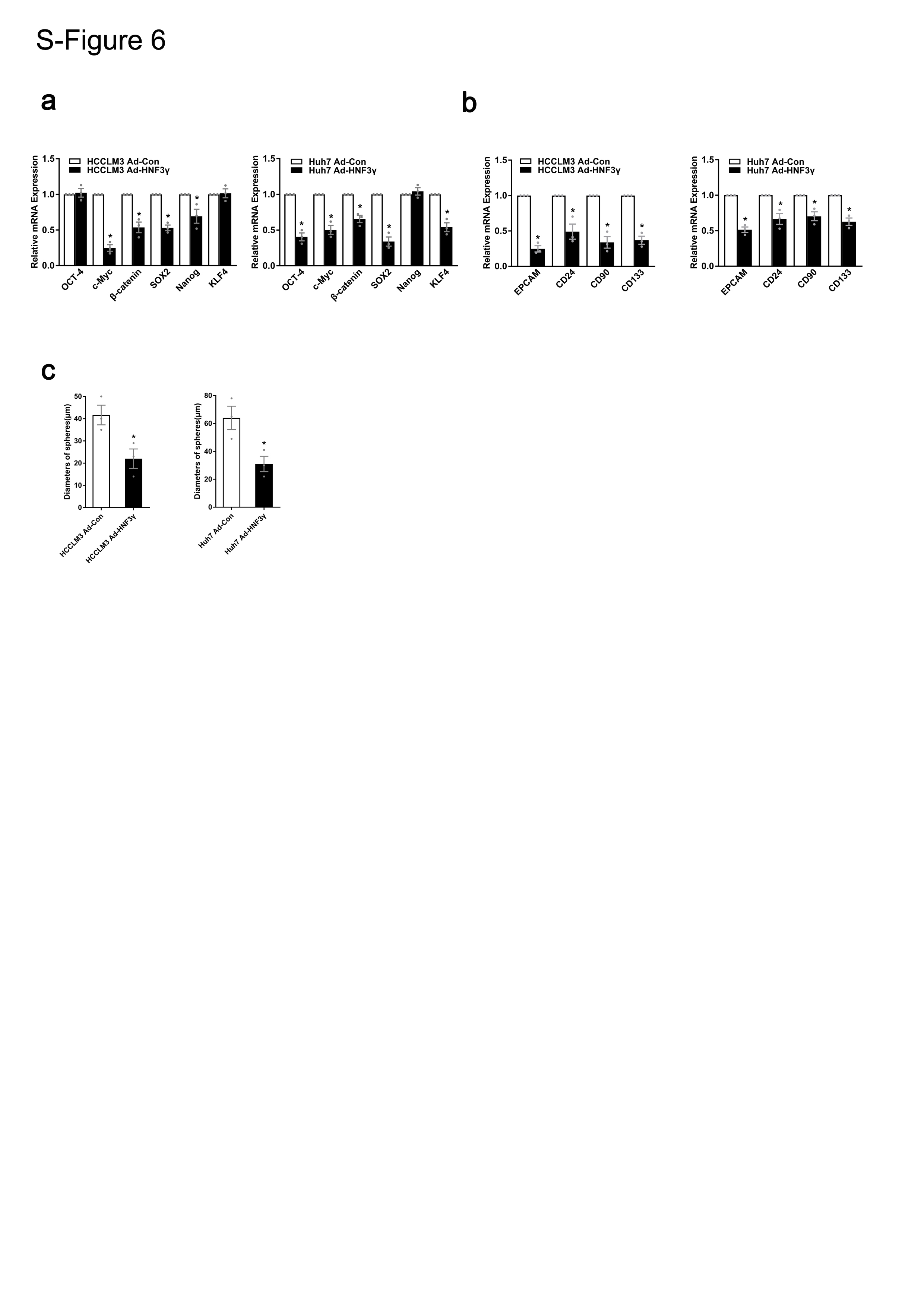
**

**Supplementary Figure 6**

a & b. The expression of stemness-associated transcription factors and liver CSC markers in HCCLM3 or Huh7 cells infected with Ad-HNF3γ or Ad-Con was analyzed by real-time PCR.

c. The diameters of spheroids formed by HCCLM3 or Huh7 cells infected with Ad-HNF3γ or Ad-Con, respectively. Results were shown as the mean ± SEM.

**
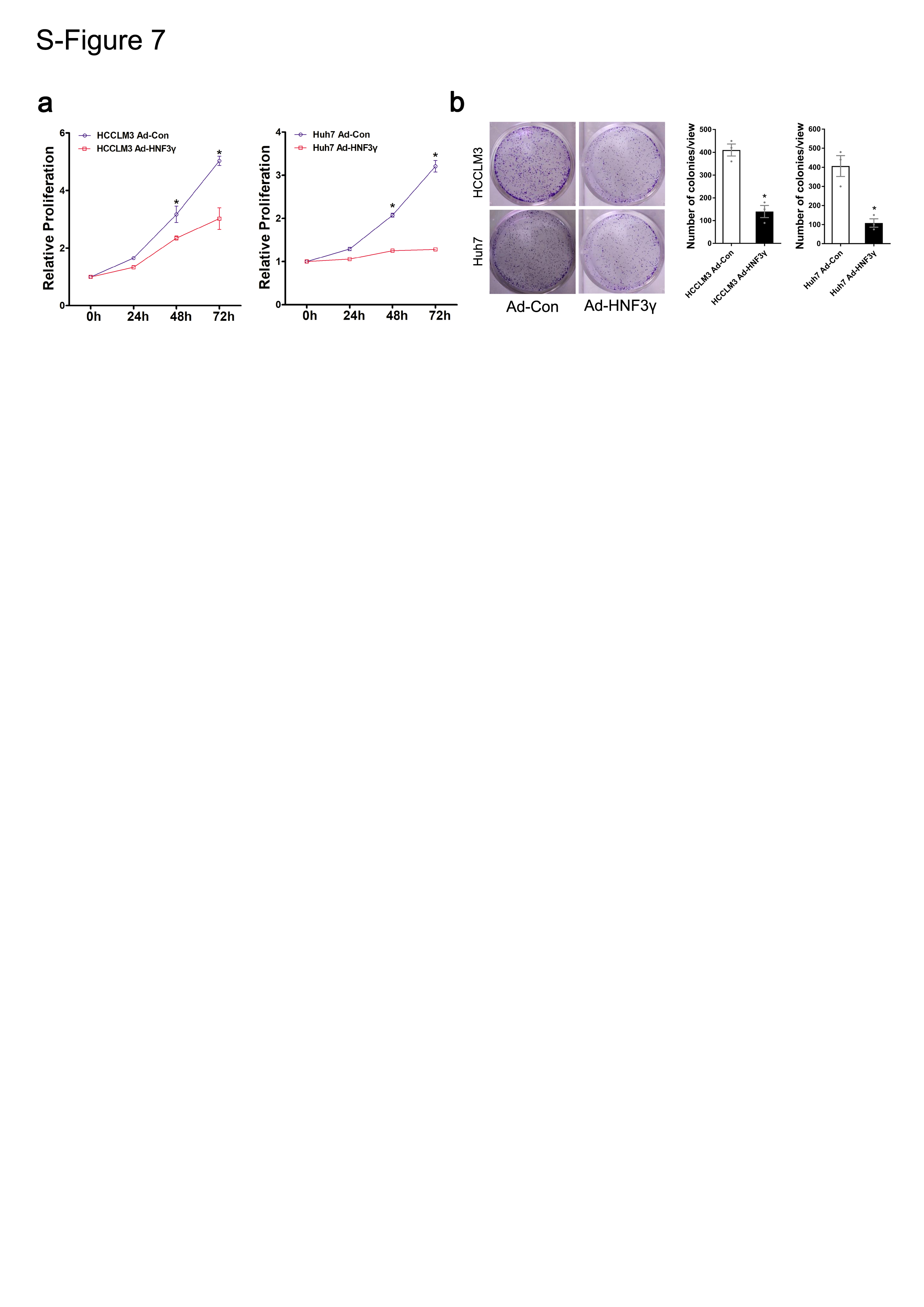
**

**Supplementary Figure 7**

a. The proliferation of HCCLM3 or Huh7 cells infected with Ad-HNF3γ or Ad-Con was measured by CCK8 assay.

b. HCCLM3 or Huh7 cells infected with Ad-HNF3γ or Ad-Con were subjected to colony growth assay. Results were shown as the mean ± SEM.

**
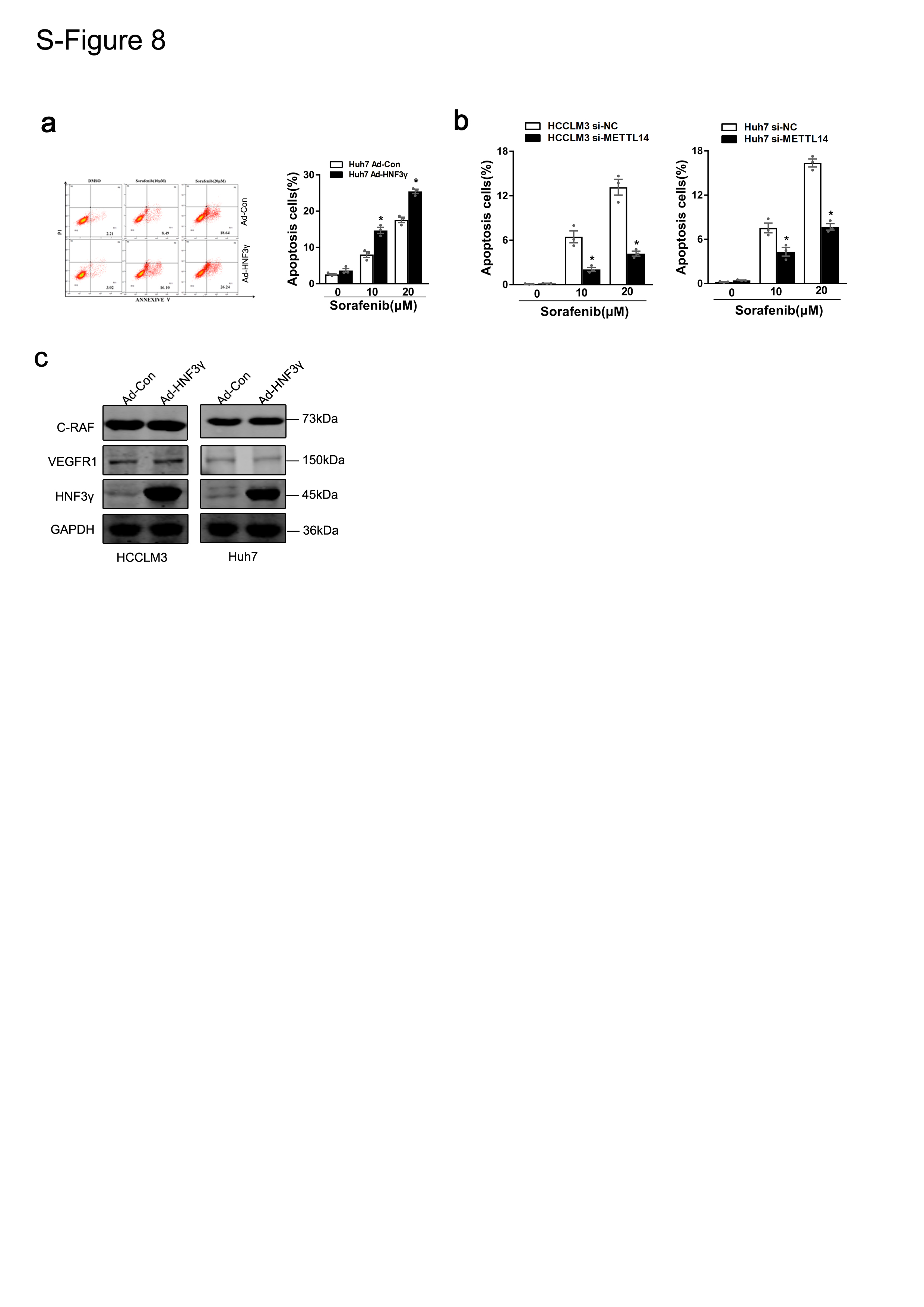
**

**Supplementary Figure 8**

a. Huh7 cells infected with Ad-HNF3γ or Ad-Con were treated with sorafenib (10 µM or 20 µM) for 24 hours, and apoptotic cells were detected by flow cytometry. Results were shown as the mean ± SEM.

b. HCCLM3 and Huh7 cells transfected with si-METTL14 or si-NC were treated with sorafenib (10 µM or 20 µM) for 24 hours, and apoptotic cells were detected by flow cytometry. Results were shown as the mean ± SEM.

c. HCCLM3 or Huh7 cells were infected with Ad-HNF3γ or Ad-Con followed by western blot assay.

**Supplementary Tables**

**Supplementary Table 1. Clinicopathological features of 156 HCCs in c**ohort 1

| Characteristics |  | HNF3γ low(75) | HNF3γ high(81) | p value |
| --- | --- | --- | --- | --- |
| AGE | ≤45 | 31(41.33%) | 26(32.10%) | >0.05 |
|  | >45 | 44(58.67%) | 55(67.90%) |  |
| Gender | male | 65(86.67%) | 69(85.19%) | >0.05 |
|  | female | 10(13.33%) | 12(14.81%) |  |
| HBsAg | + | 69(92.00%) | 70(86.42%) | >0.05 |
|  | - | 6(08.00%) | 11(13.58%) |  |
| ALB(μg/L) | ≤34 | 4(05.33%) | 4(04.94%) | >0.05 |
|  | >34 | 71(94.67%) | 77(95.06%) |  |
| ALT(μg/L) | ≤40 | 26(34.67%) | 35(43.21%) | >0.05 |
|  | >40 | 49(65.33%) | 46(56.79%) |  |
| Pathological satellite | Yes | 44(58.67%) | 42(51.85%) | >0.05 |
|  | No | 31(41.33%) | 39(48.15%) |  |
| MVI | Yes | 57(76.00%) | 59(72.84%) | >0.05 |
|  | No | 18(24.00%) | 22(27.16%) |  |
| Encapsulation | Yes | 20(26.67%) | 33(40.74%) | >0.05 |
|  | No | 55(73.33%) | 48(59.26%) |  |
| Portal vein tumor thrombus | Yes | 33(44.00%) | 22(27.16%) | <0.05 |
|  | No | 42(56.00%) | 59(72.84%) |  |
| Tumor size(cm) | ≤5 | 15(20.00%) | 26(32.10%) | >0.05 |
|  | >5 | 60(80.00%) | 55(67.90%) |  |
| Tumor number | Single | 59(78.67%) | 72(88.89%) | >0.05 |
|  | Multiple | 16(21.33%) | 9(11.11%) |  |
| BCLC | A | 28(37.33%) | 49(60.49%) | <0.05 |
|  | B or C | 47(62.67%) | 32(39.51%) |  |
| TNM | I-II | 31(41.33%) | 52(64.20%) | <0.05 |
|  | III-IV | 44(58.67%) | 29(35.80%) |  |

HBsAg, Hepatitis B virus surface Antigen;

MVI, Microvascular Invasion;

TNM, Tumor-Nodes-Metastasis;

BCLC, Barcelona Clinic Liver Cancer Staging.

**Supplementary Table 2. Univariate and multivariate analysis of patient survival in Cohort 1**

| Characteristics |  | OS（Univariate analysis） | | | |  | OS（Multivariate analysis） | | | |
| --- | --- | --- | --- | --- | --- | --- | --- | --- | --- | --- |
|  |  | HR | 95%CI | | p value |  | HR | 95%CI | | p value |
| AGE | ≤45 vs >45 | 0.924 | 0.633 | 1.349 | >0.05 |  |  |  |  |  |
|  |  |  |  |  |  |  |  |  |  |  |
| Gender | Male vs female | 0.701 | 0.393 | 1.250 | >0.05 |  |  |  |  |  |
|  |  |  |  |  |  |  |  |  |  |  |
| HBsAg | Positive vs Negative | 1.389 | 0.745 | 2.591 | >0.05 |  |  |  |  |  |
|  |  |  |  |  |  |  |  |  |  |  |
| ALB(μg/L) | ≤34 vs >34 | 1.754 | 0.815 | 3.776 | >0.05 |  |  |  |  |  |
|  |  |  |  |  |  |  |  |  |  |  |
| ALT(μg/L) | ≤40 vs >40 | 1.124 | 0.766 | 1.650 | >0.05 |  |  |  |  |  |
|  |  |  |  |  |  |  |  |  |  |  |
| Encapsulation | Yes vs No | 0.632 | 0.417 | 0.959 | <0.05 |  | 0.691 | 0.450 | 1.060 | 0.090 |
| MVI | Yes vs No | 2.012 | 1.260 | 3.215 | <0.05 |  |  |  |  | >0.25 |
|  |  |  |  |  |  |  |  |  |  |  |
| Pathological satellite | Yes vs No | 1.829 | 1.243 | 2.691 | <0.05 |  | 1.392 | 0.933 | 2.078 | 0.106 |
|  |  |  |  |  |  |  |  |  |  |  |
| Portal vein tumor thrombus | Yes vs No | 2.503 | 1.706 | 3.672 | <0.05 |  |  |  |  | >0.25 |
|  |  |  |  |  |  |  |  |  |  |  |
| Tumor size(cm) | ≤5 vs >5 | 1.698 | 1.071 | 2.693 | <0.05 |  |  |  |  | >0.25 |
|  |  |  |  |  |  |  |  |  |  |  |
| Tumor number | Single vs Multiple | 1.473 | 0.915 | 2.372 | >0.05 |  |  |  |  |  |
|  |  |  |  |  |  |  |  |  |  |  |
| BCLC | B/C vs 0/A | 2.498 | 1.697 | 3.677 | <0.05 |  | 2.140 | 1.430 | 3.201 | 0.000 |
|  |  |  |  |  |  |  |  |  |  |  |
| TNM | III-IV vs I-II | 2.379 | 1.625 | 3.482 | <0.05 |  |  |  |  | >0.25 |
|  |  |  |  |  |  |  |  |  |  |  |
| CHILD | B vs A | 1.324 | 0.767 | 2.285 | >0.05 |  |  |  |  |  |
|  |  |  |  |  |  |  |  |  |  |  |
| HNF3γ | High vs Low | 0.504 | 0.344 | 0.737 | <0.05 |  | 0.742 | 0.599 | 0.918 | 0.006 |
|  |  |  |  |  |  |  |  |  |  |  |

HBsAg, Hepatitis B virus surface Antigen;

MVI, Microvascular Invasion;

TNM, Tumor-Nodes-Metastasis;

BCLC, Barcelona Clinic Liver Cancer Staging.

|  |  |  |  |  |  |  |  |  |  |  |
| --- | --- | --- | --- | --- | --- | --- | --- | --- | --- | --- |

**Supplementary Tabl**e 3. The hepatic differentiation-associated genes upregulated in Ad-HNF3γ-infected HCC cells

| **Symbol** | **Description** | **GO Term** |
| --- | --- | --- |
| FOXN4 | forkhead box N4 | cell differentiation |
| ETV5 | ets variant 5 | cell differentiation |
| ANGPTL4 | angiopoietin-like 4 | cell differentiation |
| SMOC1 | SPARC related modular calcium binding 1 | cell differentiation |
| CBFA2T3 | core-binding factor, runt domain, alpha subunit 2; translocated to, 3 | cell differentiation |
| HNF4γ | Hepatocyte Nuclear Factor 4, Gamma | cell differentiation |
| DUSP6 | dual specificity phosphatase 6 | cell differentiation |
| HNF3γ | Hepatocyte Nuclear Factor 3, Gamma | cell differentiation |
| ELF3 | E74-like factor 3 (ets domain transcription factor, epithelial-specific) | cell differentiation |
| DLL4 | delta-like 4 (Drosophila) | cell differentiation |
| SCRIB | scribbled planar cell polarity protein | cell differentiation |
| FHL1 | four and a half LIM domains 1 | cell differentiation |
| FOXL1 | forkhead box L1 | cell differentiation |
| PMP22 | peripheral myelin protein 22 | cell differentiation |
| WNT4 | wingless-type MMTV integration site family, member 4 | cell differentiation |
| NR1D1 | nuclear receptor subfamily 1, group D, member 1 | cell differentiation |
| HLX | H2.0-like homeobox | cell differentiation |
| PAX5 | paired box 5 | cell differentiation |
| ANG | angiogenin, ribonuclease, RNase A family, 5 | cell differentiation |
| ASNS | asparagine synthetase (glutamine-hydrolyzing) | liver development |
| WNT4 | wingless-type MMTV integration site family, member 4 | liver development |
| LBP | lipopolysaccharide binding protein | liver development |
| HLX | H2.0-like homeobox | liver development |
| SLCO2B1 | solute carrier organic anion transporter family, member 2B1 | liver development |
| LSR | lipolysis stimulated lipoprotein receptor | liver development |
| FGA | fibrinogen alpha chain | liver regeneration |
| HNF4α | hepatocyte nuclear factor 4, alpha | regulation of lipid metabolic process |
| NR1D1 | nuclear receptor subfamily 1, group D, member 1 | regulation of lipid metabolic process |
| LSR | lipolysis stimulated lipoprotein receptor | regulation of lipid metabolic process |
| HNF4α | hepatocyte nuclear factor 4, alpha | lipid metabolic process |
| AKR1C2 | aldo-keto reductase family 1, member C2 | lipid metabolic process |
| PTEN | phosphatase and tensin homolog | lipid metabolic process |
| PCK1 | phosphoenolpyruvate carboxykinase 1 (soluble) | lipid metabolic process |
| GCKR | glucokinase (hexokinase 4) regulator | cellular glucose homeostasis |
| HNF4α | hepatocyte nuclear factor 4, alpha | glucose homeostasis |

**Supplementary Table 4**. Clinicopathological features of 67 HCCs in cohort 2

| Characteristics |  | HNF3γ low(32) | HNF3γ high(35) | p value |
| --- | --- | --- | --- | --- |
| AGE | ≤50 | 18(56.25%) | 17(48.57%) | >0.05 |
|  | >50 | 14(43.75%) | 18(51.43%) |  |
| Gender | male | 28(87.50%) | 27(77.14%) | >0.05 |
|  | female | 4(12.50%) | 8(22.86%) |  |
| HBsAg | + | 24(75.00%) | 31(88.57%) | >0.05 |
|  | - | 8(25.00%) | 4(11.43%) |  |
| AFP(μg/L) | ≤400 | 19(59.38%) | 29(82.86%) | <0.05 |
|  | >400 | 13(40.63%) | 6(17.14%) |  |
| ALB(μg/L) | ≤34 | 18(56.25%) | 19(54.29%) | >0.05 |
|  | >34 | 14(43.75%) | 16(45.71%) |  |
| ALT(μg/L) | ≤40 | 9(28.13%) | 5(14.29%) | >0.05 |
|  | >40 | 23(71.88%) | 30(85.71%) |  |
| Pathological satellite | Yes | 8(25.00%) | 19(54.29%) | >0.05 |
|  | No | 24(75.00%) | 16(45.71%) |  |
| MVI | Yes | 6(18.75%) | 6(17.14%) | >0.05 |
|  | No | 26(81.25%) | 29(82.86%) |  |
| Edmondson | Ⅰ-Ⅱ | 2(6.25%) | 12(34.29%) | <0.05 |
|  | Ⅲ-Ⅳ | 30(93.75%) | 23(65.71%) |  |
| Encapsulation | Yes | 15(46.88%) | 18(51.43%) | >0.05 |
|  | No | 17(53.13%) | 17(48.57%) |  |
| Portal vein tumor thrombus | Yes | 4(12.50%) | 0(00.00%) | <0.05 |
|  | No | 28(87.50%) | 35(100.00%) |  |
| Tumor size(cm) | ≤5 | 10(31.25%) | 21(60.00%) | <0.05 |
|  | >5 | 22(68.75%) | 14(40.00%) |  |
| Tumor number | Single | 29(90.63%) | 29(82.86%) | >0.05 |
|  | Multiple | 3(09.38%) | 6(17.14%) |  |

HBsAg, Hepatitis B virus surface Antigen;

AFP, α-fetoprotein;

MVI, Microvascular Invasion.

**Supplementary Table 5. Clinicopathological information of 96 patients in Cohort 3**

| **Characteristic** | **Number of patients** |
| --- | --- |
| Sex |  |
| male | 88 |
| female | 8 |
| Age (years) |  |
| ≤50 | 61 |
| >50 | 35 |
| HBV |  |
| positive | 80 |
| negative | 16 |
| ALT (μg/L) |  |
| ≤40 | 30 |
| >40 | 66 |
| Pathological satellite |  |
| yes | 35 |
| no | 60 |
| MVI |  |
| yes | 63 |
| no | 31 |
| Encapsulation |  |
| yes | 55 |
| no | 41 |
| Portal vein tumor thrombus |  |
| yes | 33 |
| no | 63 |
| Tumor size (cm) |  |
| ≤5 | 30 |
| >5 | 66 |
| Tumor number |  |
| Single | 89 |
| Multiple | 7 |
| BCLC |  |
| A | 55 |
| B or C | 41 |
| TNM |  |
| I-II | 58 |
| III-IV | 38 |
| HNF3γ |  |
| low | 49 |
| high | 47 |

MVI, Microvascular Invasion;

BCLC, Barcelona Clinic Liver Cancer Staging;

TNM, Tumor-Nodes-Metastasis.

**Supplementary Table 6. Clinicopathological information of 85 patients in Cohort 4**

| **Characteristic** | **Number of patients** |
| --- | --- |
| Sex |  |
| male | 79 |
| female | 6 |
| Age (years) |  |
| ≤50 | 55 |
| >50 | 30 |
| HBV |  |
| positive | 83 |
| negative | 2 |
| AFP (μg/L) |  |
| ≤100 | 50 |
| >100 | 35 |
| Pathological satellite |  |
| yes | 41 |
| no | 42 |
| MVI |  |
| yes | 27 |
| no | 54 |
| Portal vein tumor thrombus |  |
| yes | 20 |
| no | 64 |
| Tumor size (cm) |  |
| ≤5 | 74 |
| >5 | 11 |
| Tumor number |  |
| single | 46 |
| multiple | 39 |
| BCLC |  |
| A | 27 |
| B or C | 58 |
| TNM |  |
| I-II | 33 |
| III-IV | 52 |
| HNF3γ |  |
| low | 42 |
| high | 43 |

AFP, α-fetoprotein;

MVI, Microvascular Invasion;

BCLC, Barcelona Clinic Liver Cancer Staging;

TNM, Tumor-Nodes-Metastasis.

**Supplementary Table 7. Primers and si-RNAs l**ist

| **Gene** | **Forward primer (5’-3’)** | **Reverse primer (5’-3’)** | |
| --- | --- | --- | --- |
| HNF3γ(Human) | Forward (5*′*- 3*′*) | CTGGCCGAGTGGAGCTACTA | |
|  | Reverse(5*′*- 3*′*) | AGGGGGATAGGGAGAGCTTA | |
| β-actin(Human) | Forward (5*′*- 3*′*) | AATCGTGCGTGACATTAAGGAG | |
|  | Reverse(5*′*- 3*′*) | ACTGTGTTGGCGTACAGGTCTT | |
| 18S(Human) | Forward (5*′*- 3*′*) | CGGCTACCACATCCAAGGAA | |
|  | Reverse(5*′*- 3*′*) | GCTGGAATTACCGCGGCT | |
| Dicer (Human) | Forward (5*′*- 3*′*) | GGTGGTCCACGAGTCACAAT | |
|  | Reverse(5*′*- 3*′*) | TAGCACTGCCTTCGTTTCGT | |
| KLF4 (Human) | Forward (5*′*- 3*′*) | GCCCCTCGGGCGGCTTCGTGGCCGAGCTC | |
|  | Reverse(5*′*- 3*′*) | CGTACTCGCTGCCAGGGGCG | |
| METLL3 (Human) | Forward (5*′*- 3*′*) | TCAGCATCGGAACCAGCAAAG | |
|  | Reverse(5*′*- 3*′*) | TCCTGACTGACCTTCTTGCTC | |
| METTL14 (Human) | Forward (5*′*- 3*′*) | | GTTGGAACATGGATAGCCGC | | --- | | CAATGCTGTCGGCACTTTCA |   CTACCGTAAGTTTTTCCAAT | |
|  | Reverse(5*′*- 3*′*) | ACTACTGTACGGGTAAGCAT | |
| CD90(Human) | Forward (5*′*- 3*′*) | ATCGCTCTCCTGCTAACAGTC | |
|  | Reverse(5*′*- 3*′*) | CTCGTACTGGATGGGTGAACT | |
| EpCAM(Human) | Forward (5*′*- 3*′*) | AATCGTCAATGCCAGTGTACTT | |
|  | Reverse(5*′*- 3*′*) | TCTCATCGCAGTCAGGATCATAA | |
| CD133(Human) | Forward (5*′*- 3*′*) | AGTCGGAAACTGGCAGATAGC | |
|  | Reverse(5*′*- 3*′*) | GGTAGTGTTGTACTGGGCCAAT | |
| CD24(Human) | Forward (5*′*- 3*′*) | CTCCTACCCACGCAGATTTATTC | |
|  | Reverse(5*′*- 3*′*) | AGAGTGAGACCACGAAGAGAC | |
| OCT4(Human) | Forward (5*′*- 3*′*) | CTTGAATCCCGAATGGAAAGGG | |
|  | Reverse(5*′*- 3*′*) | GTGTATATCCCAGGGTGATCCTC | |
| SOX2(Human) | Forward (5*′*- 3*′*) | | GCCGAGTGGAAACTTTTGTCG |
|  | Reverse(5*′*- 3*′*) | | GGCAGCGTGTACTTATCCTTCT |
| Nanog(Human) | Forward (5*′*- 3*′*) | | TTTGTGGGCCTGAAGAAAACT |
|  | Reverse(5*′*- 3*′*) | | AGGGCTGTCCTGAATAAGCAG |
| c-Myc(Human) | Forward (5*′*- 3*′*) | | GGCTCCTGGCAAAAGGTCA |
|  | Reverse(5*′*- 3*′*) | | CTGCGTAGTTGTGCTGATGT |
| β-catenin(Human) | Forward (5*′*- 3*′*) | | AAAGCGGCTGTTAGTCACTGG |
|  | Reverse(5*′*- 3*′*) | | CGAGTCATTGCATACTGTCCAT |
| WTAP (Human) | Forward (5′- 3′) | | TGTGCTGTGTAAGGGCATTCGTACTCATGC |
|  | Reverse(5′- 3′) | | ACTGGGCAAACTTGGCAGTCATAAACCCAC |
| FTO (Human) | Forward (5′- 3′) | | AGGAAATCCATAATGAGG |
|  | Reverse(5′- 3′) | | TGAGGTCAAAGGGCAGAG |
| ALKBH5 (Human) | Forward (5′- 3′) | | CGGCGAAGGCTACACTTACG |
|  | Reverse(5′- 3′) | | CCACCAGCTTTTGGATCACCA |
| Albumin (Human) | Forward (5*′*- 3*′*) | | TGCACAGAATCCTTGGTGAA |
|  | Reverse(5*′*- 3*′*) | | TTCACGAGCTCAACAAGTGC |
| CYP1A2 (Human) | Forward (5*′*- 3*′*) | | CTGGCCTCTGCCATCTTCTG |
|  | Reverse(5*′*- 3*′*) | | TTAGCCTCCTTGCTCACATGC |
| PEPCK (Human) | Forward (5*′*- 3*′*) | | GTGTCCCTCTAGTCTATGAAGC |
|  | Reverse(5*′*- 3*′*) | | ATTGACTTGATCCTCCAGATAC |
| G-6-P (Human) | Forward (5*′*- 3*′*) | | GGCTCCATGACTGTGGGATC |
|  | Reverse(5*′*- 3*′*) | | TTCAGCTGCACAGCCCAGAA |
| GS (Human) | Forward (5*′*- 3*′*) | | CCTGCTTGTATGCTGGAGTC |
|  | Reverse(5*′*- 3*′*) | | GAAAAGTCGTTGATGTTGGA |
| AAT (Human) | Forward (5*′*- 3*′*) | | TATGATGAAGCGTTTAGGC |
|  | Reverse(5*′*- 3*′*) | | CAGTAATGGACAGTTTGGGT |
| MRP2 (Human) | Forward (5*′*- 3*′*) | | AGCGTCCTCTGACACTCG |
|  | Reverse(5*′*- 3*′*) | | GGCATCTTGGCTTTGACT |
| AFP (Human) | Forward (5*′*- 3*′*) | | GAACTTTCCAAGCCATAACTG |
|  | Reverse(5*′*- 3*′*) | | TACATTGACCACGTTCCAGC |
| ANGPTL4 (Human) | Forward (5*′*- 3*′*) | | GGGAGAGGCAGAGTGGACTA |
|  | Reverse(5*′*- 3*′*) | | AAACCACCAGCCTCCAGAGA |
| WNT4 (Human) | Forward (5*′*- 3*′*) | | ACACGGCCACTCAGAAAACA |
|  | Reverse(5*′*- 3*′*) | | CCAGAAGGCAGGTGGCATTA |
| HLX (Human) | Forward (5*′*- 3*′*) | | GCCTACCTCCCGACATTCAC |
|  | Reverse(5*′*- 3*′*) | | GAGACTCCCCTCTGCTTTGG |
| ELF3 (Human) | Forward (5*′*- 3*′*) | | TGAACCTGCACACTCCAGTC |
|  | Reverse(5*′*- 3*′*) | | GGTTGCTCAGGGTCAGTACC |
| LBP (Human) | Forward (5*′*- 3*′*) | | CCACCGTTCTCCAGTTACCC |
|  | Reverse(5*′*- 3*′*) | | GAAGGGTCGGAAGGACTTGG |
| SLCO2B1 (Human) | Forward (5*′*- 3*′*) | | TGGCAGAAAAGGCTGGGATT |
|  | Reverse(5*′*- 3*′*) | | CGGCTGCCAAAATAGCTCAC |
| LSR (Human) | Forward (5*′*- 3*′*) | | ACCATCACCGGAATGTATGC |
|  | Reverse(5*′*- 3*′*) | | TCCCGAAGCAGGGGTACATA |
| HNF4α (Human) | Forward (5*′*- 3*′*) | | TGCGACTCTCCAAAACCCTC |
|  | Reverse(5*′*- 3*′*) | | ATTGCCCATCGTCAACACCT |
| HNF4γ(Human) | Forward (5*′*- 3*′*) | | CACCTCCTCGCTTTCAGCAAA |
|  | Reverse(5*′*- 3*′*) | | CGGTCAAGGCAGCAATCACT |
| PMP22 (Human) | Forward (5*′*- 3*′*) | | GGGGAGGGTCTTGCCTTAAC |
|  | Reverse(5*′*- 3*′*) | | CAGCGTAACCCCTTCTTCCA |
| FOXN4 (Human) | Forward (5*′*- 3*′*) | | GACCCTGCCCTGTAACTGAC |
|  | Reverse(5*′*- 3*′*) | | AGAGGGGTGGTGATTTTGCC |
| SCRIB (Human) | Forward (5*′*- 3*′*) | | CTCTCCGAGCACAGATGGTC |
|  | Reverse(5*′*- 3*′*) | | AAAGTCCGGTGACAGGCGTC |
| PCK1 (Human) | Forward (5*′*- 3*′*) | | ATCCCCAAAACAGGCCTCAG |
|  | Reverse(5*′*- 3*′*) | | AGGGCCAGTTGTTGACCAAA |
| GCKR (Human) | Forward (5*′*- 3*′*) | | CGACAAGTAGCAGAGCGGAT |
|  | Reverse(5*′*- 3*′*) | | TCTCGGAAATCTGGTGCTGAC |
| OATP1B1 (Human) | Forward (5*′*- 3*′*) | | AGCAACAGTATGGTCAGCCT |
|  | Reverse(5*′*- 3*′*) | | GGTCATGGTTAGTCCGGCAA |
| OATP1B3 (Human) | Forward (5*′*- 3*′*) | | TTCGTGGCATAGGGGAAACC |
|  | Reverse(5*′*- 3*′*) | | AGAGATCCCAGTGCAAAGCC |
| IGF2BP1 | Forward (5*′*- 3*′*) | | CGGAGAAACGTGACACACCA |
|  | Reverse(5*′*- 3*′*) | | ACTTTCTTTGCACCCCACCC |
| IGF2BP2 | Forward (5*′*- 3*′*) | | GTGGGAGGTGTTGGATGGAC |
|  | Reverse(5*′*- 3*′*) | | GTTGACAACGGCGGTTTCTG |
| IGF2BP3 | Forward (5*′*- 3*′*) | | TTGAGCACTCGGTCCCAAAA |
|  | Reverse(5*′*- 3*′*) | | ACTATCCAGCACCTCCCACT |
| siMETTL3 | 5’-GGAGAUCCUAGAGCUAUUA-3’ | | |
| siMETTL14 | 5'-TGGTGCCGTGTTAAATAGCAA-3' | | |
| siWTAP | 5'-GGGCAAGTACACAGATCTTAA-3' | | |
| siFTO | 5’-CAGGCACCUUGGAUUAUA-3’ | | |
| siALKBH5 | 5’-CUGAGAACUACUGGCGCAA-3’ | | |
| siOATP1B1 | 5’-GCAUGCCUUUAUGGUUAATT -3’ | | |
| siOATP1B3 | 5’-GGAUAAGUCUAUGCAUCUATT-3’ | | |
| siHNF4α | 5’-GGGAACCAACGUCAUCGUUTT-3’ | | |
| siIGF2BP1 | 5’- GGCTCAGTATGGTACAGTA -3’ | | |
| siIGF2BP2 | 5’- CATGCCGCATGATTCTTGA -3’ | | |
| siIGF2BP3 | 5’- GCTGAGAAGTCGATTACTA -3’ | | |

**Supplementary Table 8. Antibodies** list

| **Antigens** | **Manufacturer** | **Application** |
| --- | --- | --- |
| HNF3γ | Santa Cruz Biotechnology, CA | 1:500 for WB |
| HNF3γ | Sigma, USA | 1:1000 for IHC |
| METTL14 | Abcam, USA | 1:100 for IHC |
| M6A | EMD Millipore, USA | For M6A RIP |
| CK19 | Servicebio, China | 1:300 for IHC |
| CD133 | Abcam, AB Biotech Company Ltd, Hong Kong | 1:100 for IHC |
| AFP | Proteintech Group, China | 1:100 for IHC |
| Ki67 | Proteintech Group, China | 1:200 for IHC |
| GAPDH | Santa Cruz Biotechnology, CA | 1:5000 for WB |
| AFP | Proteintech Group, China | 1:200 for IHC |
| PARP | CST, USA | 1:500 for WB |
| CK19 | Sigma, USA | 1:1000 for IHC |
| IGF2BP1 | Proteintech Group, China | For RIP |
| IGF2BP2 | Proteintech Group, China | For RIP |
| IGF2BP3 | Proteintech Group, China | For RIP |
